# Phytochemical Profiling and Bioactivity Assessment of *Teucrium capitatum* L. Essential Oil and Extracts: Experimental and In Silico Insights

**DOI:** 10.3390/ph17121578

**Published:** 2024-11-24

**Authors:** Redouane Tarik, Aziz Drioiche, Jalila El Amri, Mohamed Ed-Dahmouny, Abdelaaty Abdelaziz Shahat, Nadia Hadi, Mouradi Aicha, Handaq Nadia, Fadoua El Makhoukhi, Abdelhakim El Ouali Lalami, Noureddine Elmoualij, Eto Bruno, Hajji Lhoussain, Touriya Zair

**Affiliations:** 1 Research Team of Chemistry of Bioactive Molecules and the Environment, Laboratory of Innovative Materials and Biotechnology of Natural Resources, Faculty of Sciences, Moulay Ismaïl University, B.P. 11201, Zitoune, Meknes 50070, Morocco; jalilaelamri83@gmail.com (J.E.A.); simo.dahmouny@gmail.com (M.E.-D.); nadia.hadi1@gmail.com (N.H.); mouradi.aicha@umi.ac.ma (M.A.); n.handaq@umi.ac.ma (H.N.); elmakhoukhi@cnrst.ma (F.E.M.); titisfrance@gmail.com (E.B.); t.zair@umi.ac.ma (T.Z.); 2 Bio-Inorganic Chemistry, Molecular Materials and Environment, Laboratory of Innovative Materials and Biotechnology of Natural Resources, Faculty of Sciences, Moulay Ismaïl University, B.P. 11201, Zitoune, Meknes 50070, Morocco; n.elmoualij@umi.ac.ma; 3 Higher Institute of Nursing Professions and Health Techniques of Fez, Regional Health Directorate Fez-Meknes, EL Ghassani Hospital, Fes 30050, Morocco; eloualilalami@yahoo.fr; 4 Pharmacognosy Department, College of Pharmacy, King Saud University, Riyadh 11451, Saudi Arabia; ashahat@ksu.edu.sa; 5 Laboratoires TBC, Laboratory of Pharmacology, Pharmacokinetics and Clinical Pharmacy, Faculty of Pharmaceutical and Biological Sciences, P.O. Box 83, F-59000 Lille, France; 6 Bioactive, Health and Environment Laboratory, Faculty of Sciences, Moulay Ismail University of Meknes, B.P. 11201 Zitoune, Meknes 50050, Morocco; l.hajji@umi.ac.ma

**Keywords:** *Teucrium capitatum* L., β-pinene, α-cadinol, shyobunol, poliumoside, cirsimaritin, antibacterial, antifungal, antioxidant

## Abstract

**Background:** *Teucrium capitatum* L., a member of the Lamiaceae family, is widely used in traditional medicine for its therapeutic properties. This study aims to analyze the chemical composition of its essential oil and extracts, evaluate their antimicrobial and antioxidant activities, and investigate the interactions of their bioactive compounds with biological targets using in silico methods to better understand their mechanisms of action. **Methods:** Essential oil was extracted via hydrodistillation from leaves collected in Morocco, while phenolic compounds were obtained through Soxhlet and decoction extraction methods. Gas chromatography-mass spectrometry (GC-MS) was used for chemical profiling. Antimicrobial and antioxidant activities were assessed using standard methods, including DPPH, FRAP, and TAC assays. Molecular docking was conducted to explore interactions between major constituents and biological targets. **Results:** GC-MS analysis revealed significant bioactive components in the essential oil, such as β-pinene (24.5%), α-cadinol (17.02%), and shyobunol (12.13%). Extracts (hydro-ethanolic, hydro-methanolic, and aqueous via decoction) were rich in poliumoside (27.74%) and cirsimaritin (28.22%). The essential oil and extracts showed significant antimicrobial activity, particularly against *Staphylococcus aureus*, *Escherichia coli*, *Candida albicans,* and *Aspergillus niger*. Antioxidant assays confirmed strong activity. Molecular docking results supported strong interactions of major compounds with key biological targets. **Conclusions:** The high presence of phenolic and flavonoid compounds in *Teucrium capitatum* extracts contributes to their strong antimicrobial and antioxidant properties, supporting their potential for development as natural therapeutic agents.

## 1. Introduction

The therapeutic use of plants dates back thousands of years, providing critical contributions to human health through the discovery of bioactive compounds in various plant components that can be used directly for treatment or serve as precursors for modern pharmaceuticals [1]. Particularly used in developing nations, medicinal plants are a primary source of healthcare due to their accessibility and efficacy in disease prevention and treatment [2]. Traditional medicine relies extensively on these plants for their therapeutic attributes, particularly to relieve symptoms, prevent infections, or bolster the body’s immune defenses [3].

The Lamiaceae family, also known as Labiatae, is one of the most comprehensively studied families in medicinal botany, recognized for its applications in both traditional and modern medicine. This group of aromatic plants is valued for its pharmacological potential as well as its applications in the cosmetic and culinary fields [4]. Among the genera within this family, *Teucrium* stands out, comprising over 340 species predominantly found in the Mediterranean and temperate regions [5]. These species have been used medicinally for more than two millennia, especially in treating gastrointestinal, inflammatory, and metabolic disorders [6].

However, despite their long history of medicinal use, Teucrium species are associated with significant hepatotoxicity, which has raised safety concerns regarding their use. Germander extracts, including those derived from *T. capitatum*, are known to contain terpenoids derived from neo-clerodane diterpenes, which are metabolized in the liver into epoxide derivatives via a CYP3A4-catalyzed reaction. These epoxides can induce midzonal hepatocyte necrosis and, in severe cases, lead to fulminant hepatitis or death [7,8]. Consequently, the use of germander extracts is banned in pharmaceutical applications in Europe and the USA (except for limited use in alcoholic beverages). This toxicological profile necessitates extreme caution in the traditional and therapeutic use of these plants.

*Teucrium capitatum*, or golden germander, is distributed across the dry and semi-arid areas of the Mediterranean basin, North Africa, and Western Asia [9]. Known for its notable morphological diversity, this species is used in various traditional medicine practices, notably for stomach discomfort, ulcers, and diabetes [10]. In Morocco, *T. capitatum* leaves are commonly prepared in decoction as a traditional remedy for diabetes and as a therapeutic agent [11,12].

Phytochemical analyses of various *Teucrium* species have identified a complex array of secondary metabolites, including polyphenols, flavonoids, saponins, and terpenoids [13,14]. These metabolites are responsible for the diverse biological properties attributed to these species, including antibacterial, antioxidant, anti-inflammatory, and antidiabetic activities [15,16]. The essential oil of *T. capitatum* is particularly rich in bioactive compounds, notably sesquiterpenes like caryophyllene and germacrene D, which exhibit anti-inflammatory and antibacterial effects, as well as antioxidant capacity. Additionally, the monoterpenes α-pinene and β-pinene are recognized for their antimicrobial properties, with α-pinene also functioning as a bronchodilator and β-pinene showing antibacterial and antifungal properties [17,18]. Nonetheless, the hepatotoxicity associated with certain sesquiterpenes and neo-clerodane derivatives must be taken into account when considering the therapeutic applications of *T. capitatum*.

Debbabi et al. [19] reported a high concentration of monoterpenes and sesquiterpenes in *T. capitatum*, compounds known for their antibacterial and antifungal activities. Studies on the *Teucrium* species have demonstrated a range of pharmacological properties, including diuretic, antipyretic, antispasmodic, and anti-inflammatory effects [20,21,22,23]. These species are rich in sesquiterpenes and monoterpenes, such as caryophyllene and α-pinene, which are celebrated for their antioxidant, antimicrobial, and anti-inflammatory properties, alongside flavonoids such as apigenin, luteolin, and quercetin, known for their potent antioxidant effects [24,25]. Bioactive phenols like carvacrol further enhance the therapeutic potential of these species for both medicinal and cosmetic applications [26].

Despite extensive studies on the *Teucrium* species, *T. capitatum* remains underexplored. Preliminary investigations, however, have revealed promising antibacterial and antioxidant properties. Therefore, a more detailed characterization of its essential oils and extracts is warranted, as well as an examination of their interactions with specific biological targets to elucidate their mechanisms of action.

In this context, in silico approaches, like molecular docking, provide an effective means of simulating interactions between bioactive compounds and target proteins. Molecular docking enables the prediction of binding affinities and interaction mechanisms, supporting the identification of promising compounds for therapeutic development [27]. This study will use molecular docking to investigate the interactions between the major constituents of *T. capitatum* essential oils and proteins linked to antibacterial and antioxidant activities. This methodology is anticipated to offer mechanistic insights that could guide future research and support the development of new therapeutic agents from natural sources.

The primary aim of this research is to enhance the understanding of *T. capitatum* essential oils (EOs) by analyzing their chemical composition and evaluating their biological activities, including antibacterial, antifungal, and antioxidant properties. Additionally, in silico molecular docking will be used to investigate possible interactions between the bioactive compounds and biological targets, providing a mechanistic insight into their therapeutic potential.

## 2. Results and Discussion

### 2.1. Quality Control Study of T. capitatum

The quality control of the plant material of *T. capitatum* was carried out by determining the moisture content (MC), pH, titratable acidity, and ash content. The results obtained are presented in Table 1.

The moisture content of medicinal plants is a crucial indicator for verifying their proper storage and handling. According to the standards of the European Pharmacopoeia, this content should not exceed 12% (Table 1). The results of the present study show that the powder used meets these standards, with an average value of 10.02 ± 1.69%. The hydrogen potential (pH) is also an important parameter for assessing a plant’s capacity to absorb and deliver nutrients. The pH of *T. capitatum* is estimated at 5.98 ± 0.01, classifying it among acidophilic plants, which contain no total limestone, within a range of 4.0 to 6.5. Regarding titratable acidity, the *T. capitatum* powder shows an acidity of 0.33 ± 0.01 (Table 1), thereby measuring the quantity of free organic anions in the tested solution. Finally, the ash content, which reflects the quantity of non-volatile mineral compounds at high temperatures, is notably high for this plant, with a value of approximately 87.80 ± 0.11%.

### 2.2. Analysis of the Mineral Composition ICP-AES of Studied Plants

The determination of mineral content, including microelements (Fe and Cu), and heavy metals (As, Pb, Zn, Co, and Al), was carried out in the leaves of *T. capitatum* L.; the results are presented in Table 2. The *T. capitatum* sample revealed levels that were below the FAO/WHO-required limits.

This studied species can be considered a source of essential minerals, as it contains iron (Fe) at 7.414 mg/L, aluminum (Al) at 4.246 mg/L, zinc (Zn) at 1.688 mg/L, and manganese (Mn) at 1.231 mg/L. Copper (Cu) and arsenic (As) are present only in trace amounts, while cobalt (Co) is undetectable. While these mineral concentrations are below the FAO/WHO safety limits, further toxicological studies would be needed to conclusively confirm the plant’s non-toxicity, particularly with regard to the potential accumulation of heavy metals.

Some heavy metals (Fe, Cu, and Zn) are essential for plants and animals [28]. Metals such as Cu, Zn, Fe, Mn, Mo, Ni, and Co are imperative micronutrients [29], whose absorption over the plant’s needs can produce toxic effects [30,31]. They are also called trace elements due to their presence in trace (10 mg/kg, or mg/L) or ultra-trace (1 μm/kg, or μm/L) quantities in environmental matrices.

Essential heavy metals, including copper (Cu), zinc (Zn), iron (Fe), manganese (Mn), and molybdenum (Mo), serve critical biochemical and physiological roles in plants. They participate in redox reactions and are integral components of various enzymes. Copper, for instance, is crucial for higher plants and algae, especially in photosynthesis [32]. Zinc is essential for maintaining ribosome integrity, supporting carbohydrate synthesis, and catalyzing oxidation processes in plants; it also provides structural support for numerous transcription factors and acts as a cofactor in RNA polymerase [33]. Iron, which is indispensable for all organisms, is vital in many metabolic processes. It is an essential element found in heme-containing proteins, such as hemoglobin, myoglobin, and cytochromes, as well as in various non-heme iron proteins involved in crucial metabolic pathways. Both iron and copper function within proteins that catalyze redox reactions, playing indispensable roles in cellular metabolism [33].

In contrast, non-essential heavy metals such as arsenic (As), lead (Pb), cadmium (Cd), mercury (Hg), and antimony (Sb) have no recognized biological role and are considered toxic. The uptake of these metallic elements by plants is influenced by several factors, including soil composition, plant species, interactions between elements, and climatic conditions [34].

While essential metals like Cu, Zn, and Fe may theoretically contribute to antioxidant functions within the plant, our study’s observed antibacterial and antioxidant activities are primarily attributed to bioactive compounds such as β-pinene, α-cadinol, and flavonoids, which are well-established for these properties. Establishing a direct relationship between metal content and the bioactivity observed here would require isolating and testing these metals separately, which was outside the current study’s scope. The mineral composition analysis in this study provides a comprehensive chemical profile of *Teucrium capitatum* and contributes essential safety data, establishing a baseline for future studies exploring potential synergistic effects between these metals and other bioactive compounds.

### 2.3. Phytochemical Screening

The preliminary phytochemical screening of *Teucrium capitatum* leaves was conducted to identify primary and secondary metabolites using precipitation and coloration reactions specific to each compound family. This qualitative approach allowed us to characterize the presence and abundance of various bioactive compounds based on the intensity of observed colorations and precipitates. The results obtained, presented in Table 3, are based on our direct analysis.

The data reveal a notable richness in primary metabolites in *T. capitatum* leaves, with a high abundance of lipids, followed by proteins, as confirmed by the xanthoproteic reaction, and moderate amounts of reducing sugars and polysaccharides. Regarding secondary metabolites, we identified a significant presence of catechic tannins and a moderate amount of gallic tannins, as well as flavonoids (including flavones and leucoanthocyanins), mucilage, and saponins. However, no alkaloids (tested via Mayer, Dragendorff, and Wagner reactions) or anthocyanins were detected.

These findings suggest that *T. capitatum* could exhibit various biological activities attributed to the identified compounds. For instance, tannins and flavonoids, often associated with antioxidant and antimicrobial properties, as well as saponins and mucilage, known for their emollient and anti-inflammatory effects, contribute to the bioactive potential of the extract. This screening thus provides a robust foundation for considering *T. capitatum* as a promising natural source of compounds for future pharmacological investigations.

### 2.4. Study of the Diversity of Volatile Chemicals in T. capitatum

#### 2.4.1. Yield and Quality Control of EOs from *T. capitatum*

The EOs yield of *T. capitatum* L. was evaluated at 0.72% (*v*/*w*). This result is higher than that reported by Antunes et al. [35], who obtained a yield of 0.15% (*v*/*w*) for several populations of *T. capitatum* in Portugal. Conversely, studies such as those by Kerbouche et al. [36], recorded a similar yield of 0.3% (*v*/*w*) for *T. polium* subsp. *capitatum* in the Lakhdaria region. Comparable values have also been reported by Cozzani et al. [37] with 0.6%, De Martino et al. [38] with 0.7%, and Djabou et al. [39], with yields ranging from 0.49 to 0.62%.

The quality control of EOs is based on several physicochemical indicators, including density and organoleptic characteristics such as color and odor, as presented in Table 4. The density of *T. capitatum* EOs is 0.962 g/mL, a value consistent with the standards provided by the FAO and WHO, which range between 0.896 and 0.910 g/mL. The oil’s color is yellow-orange, and its odor is described as strong and pronounced. The density of the oil is an essential criterion for verifying the quality and conformity of the EOs with international standards, indicating alignment with prevailing standards.

#### 2.4.2. Chemical Profiling of *T. capitatum* Using GC-MS

**Figure 1:**
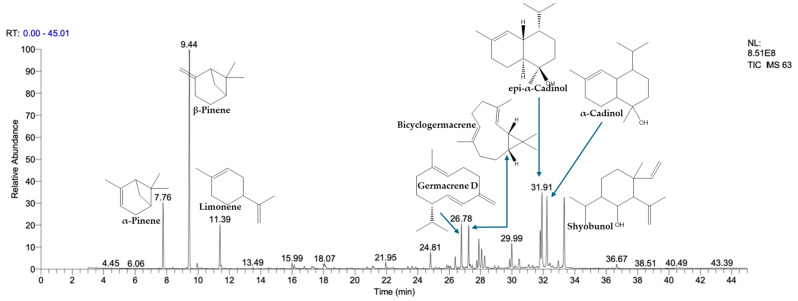
Normalized chromatographic profile from the GC-MS analysis of the studied *T. capitatum*.

The gas chromatography (GC/MS) analysis of the EOs extracted from the leaves of *T. capitatum* L., presented in Figure 1, reveals a particularly rich and diverse chemical composition. A total of 31 chemical compounds were identified, representing approximately 99.62% of the EOs’s total composition. Among these, thirteen compounds are present at concentrations above 1% (Table 5), with the most abundant being β-pinene (24.50%), followed by α-cadinol (17.02%), shyobunol (12.13%), α-pinene (8.46%), and germacrene D (4.94%). The EOs are mainly composed of three major chemical families: oxygenated sesquiterpenes (43.43%), monoterpenes (36.42%), and sesquiterpenes (17.83%), with a minor proportion of oxygenated monoterpenes (1.26%). These findings highlight the complexity and chemical richness of *T. capitatum* EOs, making it potentially valuable for applications in the therapeutic, cosmetic, and industrial sectors.

Our research reveals considerable regional diversity in the chemical composition of *T. capitatum* compared to earlier studies. In Greece, the primary ingredients detected are carvacrol (10.1%), β-caryophyllene, and germacrene D [40], but in Portugal, the concentrations of α-pinene and sabinene exhibit significant variability between samples, ranging from 0.7% to 7.7% and from 1.1% to 11.2% [35], respectively. In Serbia, germacrene D (31.8%) predominates in *T. polium*, a similar species, with β-caryophyllene and linalool [41], whereas in Iran, caryophyllene oxide (25.9%) and α-cadinol (46.2%) are the most prevalent [42].

The findings of this research exhibit a notable resemblance to those documented in Algeria. The samples examined by El Amri et al. [43] and Maccioni et al. [18] also demonstrate a preponderance of β-pinene and germacrene D, aligning with our findings. Nonetheless, several investigations exhibit significantly distinct profiles, as shown by the research conducted by Khani and Heydarian [42] in Iran, whereby the composition is mostly characterized by α-cadinol and γ-muurolene. This prior research underscores significant discrepancies, highlighting the impact of regional and environmental variables.

Additionally, it is significant that two separate chemotypes of *T. capitatum* have been recognized in certain locations. In Italy, a chemotype prevalent in coastal regions is characterized by limonene, α-pinene, and (E)-nerolidol, whilst another chemotype from the mountainous parts of Sardinia is distinguished by a high concentration of trans-α-bergamotene and humulene epoxide II [18]. This demonstrates the significant impact of growing circumstances and genetic variables on the chemical makeup of the EOs.

The chemical heterogeneity in the EOs of *T. capitatum* may be ascribed to several causes, including genetic diversity, distillation or extraction techniques, and local edaphic and climatic circumstances [25]. Boulila et al. [44] established that these factors substantially affect the composition of Tunisian EOs, corroborated by Maccioni et al. [18] in Italy, which demonstrates a pronounced variation in compositions among the Sicilian regions of Noto Antica (Syracuse), Contrada Quacella (Madonie Mountains), and Marianopoli (Caltanissetta).

Ultimately, our findings underscore the need to account for these parameters when assessing the biological characteristics of the essential oils of *T. capitatum*. The variety of chemical constituents, especially oxygenated monoterpenes, and sesquiterpenes, equips these essential oils with potential uses in diverse sectors, including medicines as antimicrobial and antioxidant agents, as well as in cosmetics and flavoring industries.

#### 2.4.3. Distribution of Major Chemical Families of the EO of *T. capitatum*

**Figure 2:**
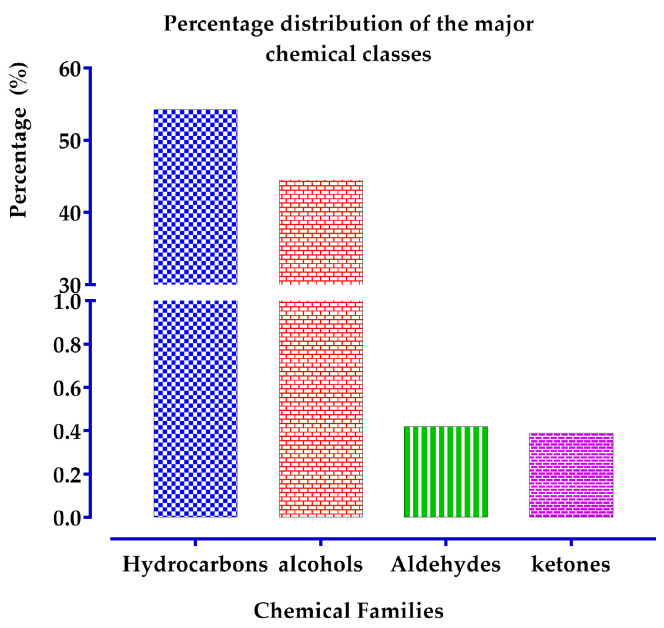
Distribution of major chemical families of the EO of *T. capitatum*.

The chemical composition of the EOs derived from *T. capitatum* L. is predominantly composed of hydrocarbons (54.29%) and monoterpene alcohols (44.56%), as illustrated in Figure 2. Monoterpene aldehydes and ketones are present in much smaller quantities, with concentrations of 0.42% and 0.39%, respectively. This distribution highlights the complexity and diversity of the chemical constituents within the oil.

Essential oils have historically been integral to therapeutic techniques due to their extensive array of biological activity. Notwithstanding progress in synthetic chemistry and the rise of the pharmaceutical, food, and cosmetic sectors, the therapeutic use of essential oils has continued to be substantial. They are regarded as a significant source of essential bioactive compounds, providing actions including anti-inflammatory, antiseptic, antiviral, deodorizing, insecticidal, and antioxidant activities [45,46]. Furthermore, a single essential oil might demonstrate many therapeutic effects concurrently, making it a multifaceted natural product with uses in diverse fields.

The many chemical constituents of EOs demonstrate a spectrum of biological activity, with each imparting unique medicinal attributes. Acids are effective anti-inflammatory agents recognized for their soothing effects on the neurological system, and aldehydes have anti-inflammatory and soothing qualities, while also being acknowledged for their anti-infectious effects. Nonetheless, these substances may irritate the skin and mucous membranes if not used cautiously. Ketones exhibit a variety of activities, including anti-inflammatory, anti-infectious, and immune-stimulating actions at low concentrations. They are renowned for their therapeutic and soothing qualities. Nonetheless, ketones may exhibit neurotoxicity at elevated levels, and therefore essential oils abundant in these chemicals should be used judiciously and not in isolation. Esters are proficient in reestablishing equilibrium within the neurological system, whilst monoterpenes are esteemed for their immune-enhancing, antibacterial, and analgesic attributes. Monoterpenes may be detrimental to the skin, possibly resulting in significant burns, hence requiring regulated application. Phenols are potent anti-infective drugs, effective against a wide range of microorganisms, fungi, viruses, and bacteria. Although phenols are very effective, they are also recognized for their irritating effects on the skin and mucous membranes, with the capacity to induce burns. In large quantities, they may damage the liver by obliterating hepatic cells. Finally, sesquiterpenes have anti-inflammatory and anti-allergic characteristics and are extensively used in cosmetology due to their dermal compatibility. They are often well-tolerated and provide therapeutic advantages in several applications.

### 2.5. Extraction and Quantitative Analysis of Phenolic Compounds

#### 2.5.1. Extraction Yield

**Figure 3:**
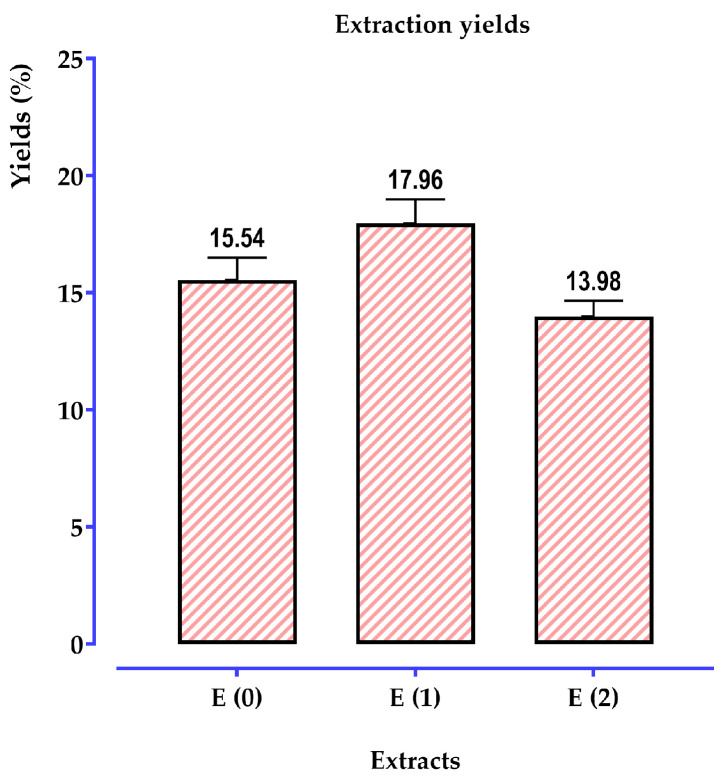
Average yields of *T. capitatum* Extracts.

The findings illustrated in Figure 3 reveal intriguing variations in extraction yields based on the method and solvents used. Specifically, the Soxhlet extraction delivered yields of (17.960 ± 0.735%) for the hydroethanolic extract and (15.537 ± 0.297%) for the aqueous extract obtained via decoction. Interestingly, the hydromethanolic extract displayed a slightly lower yield at (13.982 ± 0.078%). These variations highlight the substantial impact of both the extraction technique and solvent type on the efficiency of compound recovery. Such differences emphasize the importance of selecting an appropriate extraction approach tailored to the desired output.

The hydroethanolic yield of 17.96% aligns closely with findings from previous studies, such as that of Sharififar et al. [47], adding validation to our methodology. However, it is worth noting that the ethanolic extraction conducted by Malki et al. [48] on *T. polium* from the Biskra region reported a much higher yield of 47.76%. This striking disparity might be attributed to differences in plant material, geographic factors, or the specific extraction conditions employed, underscoring the complex interplay between biological and methodological variables. As suggested by Osorio-Tobón et al. [49], extraction yields are strongly influenced by the choice of solvent and the conditions under which extractions are performed. The results from our study reflect this, highlighting the importance of a deliberate approach to extraction to optimize yield and achieve consistency in outcomes. Such insights can guide future research towards more effective extraction methodologies, maximizing the recovery of valuable compounds from botanical sources.

#### 2.5.2. Determination of Polyphenols

**Figure 4:**
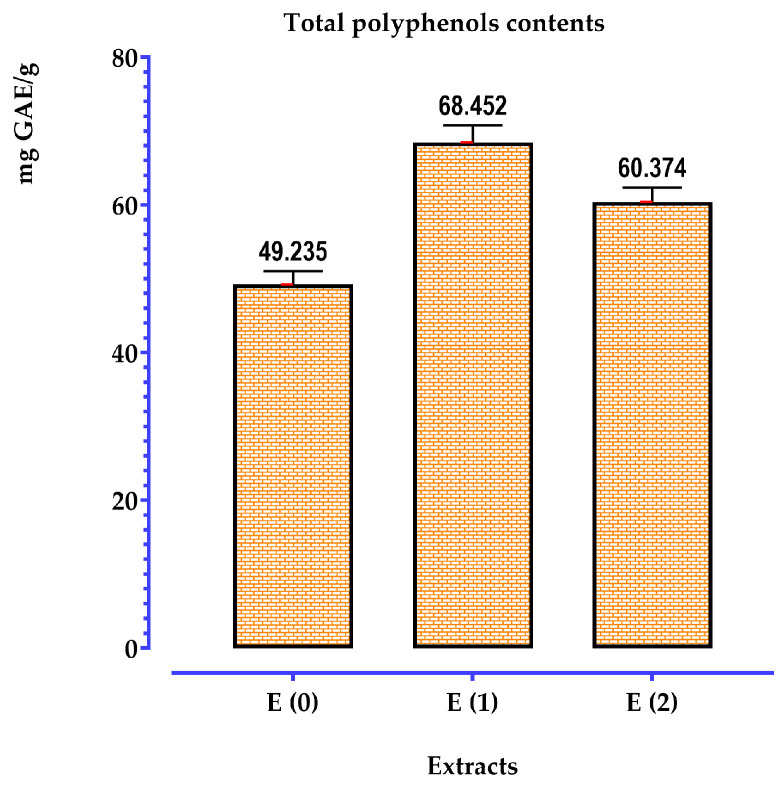
Polyphenol content of extracts from the species *T. capitatum*.

Phenolic compounds were quantified using a linear calibration curve (y = ax + b), with standard solutions of gallic acid at various concentrations. The total percentage of polyphenols in each extract was determined by utilizing the calibration curve, expressed in terms of milligrams of gallic acid equivalents per gram of extract. Optical density measurements were conducted at a wavelength of 760 nm. Concerning the phenolic content obtained, a clear variation was observed depending on the solvent used. The results indicate that the quantity of phenolic compounds ranged from 49 to 68 mg GAE/g of extract (Figure 4). The highest concentration was found in the ethanolic extract (68.452 mg GAE/g of extract), followed by the methanolic extract (60.374 mg GAE/g of extract), while the aqueous extract exhibited the lowest concentration (49.235 mg GAE/g of extract).

In a study conducted in Algeria by Djeridane and colleagues [50], a total phenolic compound content of 4.92 ± 0.21 (mg GAE/g of plant leaf) was observed in the hydro-ethanolic extract of the same plant, which is extremely low compared to our results. The total phenolic content in the methanolic extract of T. polium, a related species from Tunisia, was 34.7 ± 0.01 mg of gallic acid per mL of extract, according to Khadhri et al. [5]. A value of 48.88 ± 0.01 mg GAE/g of extract from a plant harvested in Tunisia was reported by Bakari and colleagues [51]. According to El Atki et al. [11], the total phenol content in a plant harvested in Morocco was found to be 109.26 mg GAE/g. Chabane et al. [52], reported this value as 86.63 ± 0.03 mg GAE/g.

The choice of extraction solvent can impact the levels of phenolic compounds. According to Djeridane et al. [50], the methanolic extract contains a higher amount of polyphenols than the hydro-ethanolic extract.

The origin of the plant and the climatic conditions also promote the biosynthesis of secondary metabolites such as polyphenols [53]. Secondary metabolites can vary during the growth of the plant. This may be attributed to genetic factors, soil quality, and climatic conditions (temperature, sun exposure, drought, and salinity) [54].

Our results are supported by research conducted by Bendjabeur et al. [55] and El Amri et al. [43], which demonstrate that the use of ethanol in combination with water allows for a more effective extraction of total polyphenols. According to Atik and Mohammedi [56], adding water to organic solvents improves the solubility of polyphenols by modifying the polarity of the organic solvent. According to Sripad et al. [57], this increase can be attributed to the weakening of hydrogen bonds in aqueous solutions, as well as to the increased basicity and ionization of polyphenols in these solutions. The solubility of polyphenols is primarily influenced by the number of hydroxyl groups, molecular weight, and the carbon chain length of the basic skeleton [56].

#### 2.5.3. Determination of Flavonoid

**Figure 5:**
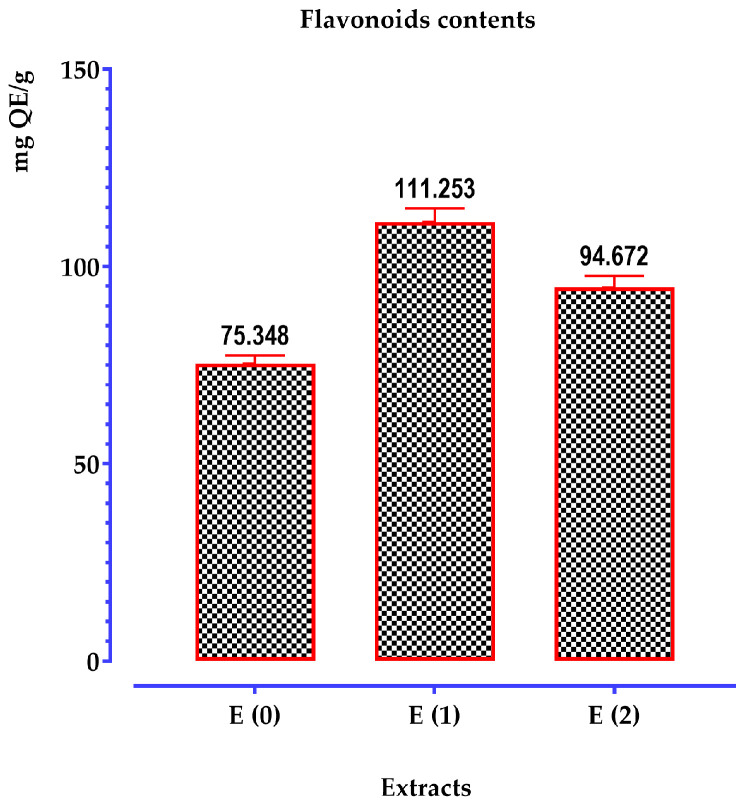
Flavonoid content of extracts from the species *T. capitatum*.

The determination of flavonoid content was performed using the aluminum chloride (AlCl_3_) method, with a linear calibration curve (y = ax + b), and prepared with standard quercetin solutions at various concentrations. The flavonoid content of each extract was calculated from the calibration curve and expressed in mg quercetin equivalents per gram of extract (mg QE/g of extract). The optical density was measured at a wavelength of 510 nm. The values from this table are represented as a histogram in Figure 5.

Based on these results, we observe that the quantity of flavonoids ranges between 75 and 111 mg QE/g of extract. The highest flavonoid content was detected in the hydro-ethanolic extract, with 111 ± 5.00 mg QE/g of extract, followed by the hydro-ethanolic extract with 91 ± 5.00 mg QE/g of extract. In contrast, the amount recorded for the aqueous extract (31.55 ± 3.00 mg QE/g of extract) remains the lowest.

According to the literature data, we can note that the total flavonoid content varies for the same plant depending on the extraction solvent and the region of origin. In this context, Djeridane et al. [50] reported a total flavonoid content of 4.63 ± 0.10 mg rutin equivalent/g dry plant of *T. polium*. Similarly, Khadhri et al. [5] observed that the total flavonoid content in the methanolic extract of a Tunisian *T. polium* is 2.67 ± 0.05 mg rutin equivalent/mL of extract. These levels remain low compared to our results. Bakari et al. [51] reported a similar value for a plant also from Tunisia (2.75 ± 0.01 mg QE/g of extract). On the other hand, Chabane et al. [52] found a value of 24.43 ± 0.01 mg QE/g. El Atki et al. [11] found a flavonoid value of 102.99 mg rutin equivalent per gram of Teucrium sp. from Morocco.

#### 2.5.4. Determination of Condensed Tannin

**Figure 6:**
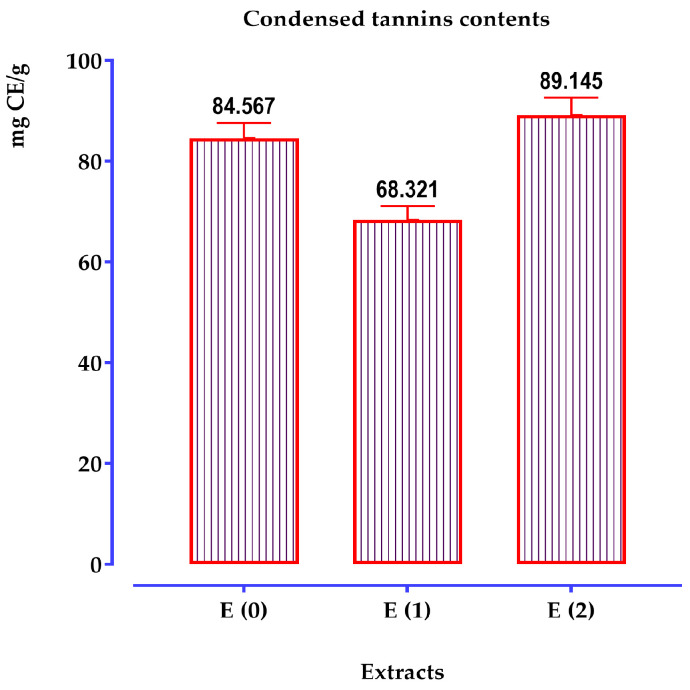
Condensed tannin content of *T. capitatum* extracts.

The condensed tannin content of the studied species was determined using the vanillin reagent and a linear calibration curve (y = ax + b) prepared with standard catechin solutions at various concentrations. The condensed tannin content of each extract was calculated from the calibration curve and expressed in mg catechin equivalents per gram of extract (mg CE/g extract). The optical density was measured at a wavelength of 550 nm, and the results are presented in Figure 6.

According to the results shown in the histogram, we note that the quantity of condensed tannins varies between 68 and 89 mg CE/g of extract. It is highest at 89 ± 4.00 mg CE/g of extract in the hydro-methanolic extract, followed by the hydro-ethanolic extract at 84 ± 6.00 mg CE/g of extract. However, it is lower in the aqueous extract, with 68 ± 3.00 mg CE/g of extract.

### 2.6. Analysis and Identification of Polyphenols in T. capitatum Extracts by High-Pressure Liquid Chromatography-Mass Spectrometry (HPLC/UV-ESI-MS)

**Figure 7:**
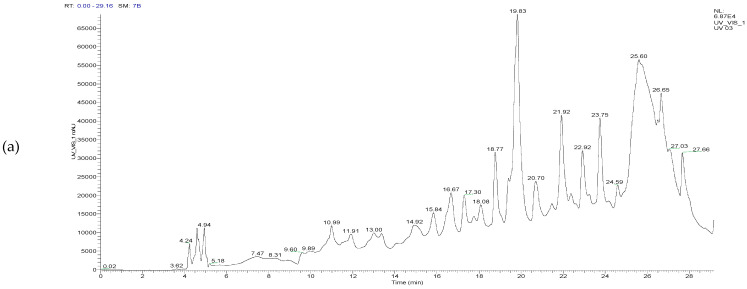

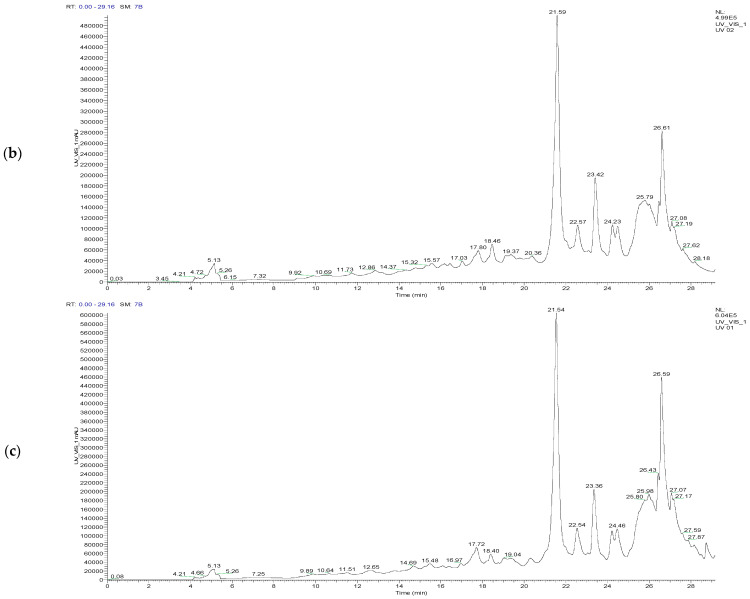
HPLC/UV-ESI-MS chromatograms of *T. capitatum* compounds: (**a**) decocted; (**b**) hydroethanolic extract; (**c**) hydromethanolic extract.

The chromatographic profile, illustrated below (Figure 7), shows the peaks of compounds from the *T. capitatum* extracts, along with their retention times and relative abundances.

The chromatographic profile of the *T. capitatum* extracts, presented in Figure 7, highlights the peaks corresponding to various compounds, as well as their retention times and relative abundances. The HPLC/UV-ESI-MS analysis allowed the identification of 66 distinct molecules belonging to several chemical classes, as detailed in Table 6.

Table 6 enumerates the compounds identified via various extraction methods, indicating that flavonoids constitute the predominant class, with respective proportions of 45.91% in the decoction, 58.56% in the hydroethanolic extract, and 57.55% in the hydromethanolic extract. Significant quantities of phytochemicals, including cirsimaritin (28.22% in the decoction) and naringenin-7-O-glucoside (15.93% in the hydroethanolic extract), were identified, substantiating the antioxidant potential of *T. capitatum* attributable to its abundance of flavonoids. These compounds are recognized for their potent antioxidant capabilities, rendering these extracts very pertinent for medicinal applications [58,59].

Table 7 demonstrates that flavonoids and phenylethanoid glycosides are the primary chemical classes in *T. capitatum*, with their quantities affected by the extraction solvent used. Decoction improves the extraction of iridoids and vitamins, but hydroethanolic extracts, including those produced using Soxhlet extraction, are more efficient at extracting flavonoids and phenylethanoid glycosides. The variations in the distribution of bioactive substances highlight the significance of solvent choice for certain therapeutic uses.

**Figure 8:**
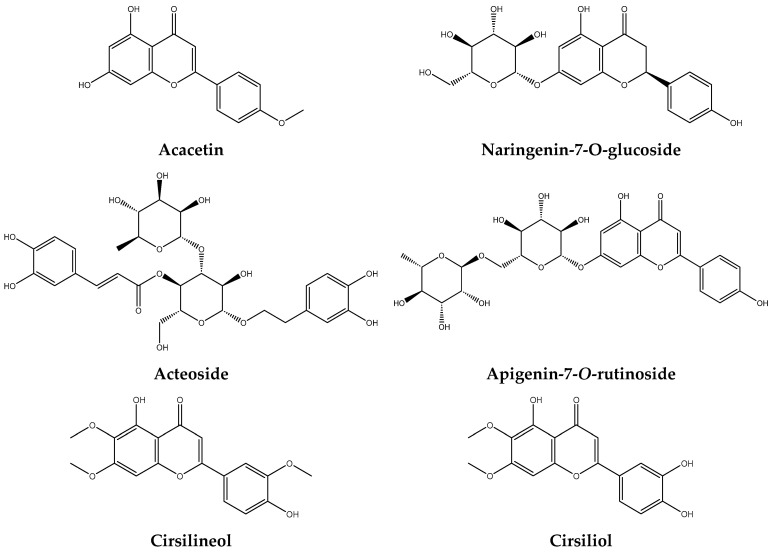

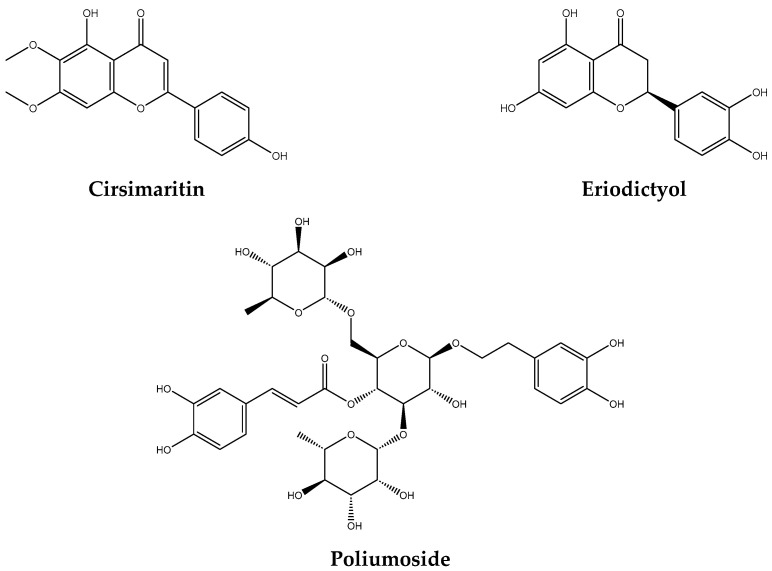
Chemical structures of principal identified compounds.

Figure 8, representing the chemical structures of the main identified compounds, complements this analysis by providing precise structural information.

The HPLC/UV-ESI-MS analysis of *T. capitatum* extracts facilitated the accurate identification of the chemicals present and yielded significant insights into their molecular structure via ion fragmentation in both negative and positive modes. This approach emphasized the distinct fragmentation spectra of the molecules, therefore aiding their identification and structural characterization.

Flavonoids, being the primary class of chemicals, are essential to the bioactivity of *T. capitatum*. Their elevated content in the diverse extracts underscores their substantial contribution to the plant’s pharmacological activities. Cirsimaritin, a predominant flavonoid in the decoction (28.22%), has shown anticancer and anti-inflammatory properties in prior research [60]. This finding corresponds with the research of Belarbi et al. [61], who also documented elevated flavonoid levels in species of the genus *Teucrium*.

In addition to flavonoids, phenylethanoid glycosides, particularly poliumoside, were particularly abundant in the extracts. Poliumoside was found in significant quantities in the hydroethanolic extract (30.63%), with a molecular weight of 770 Da, presenting an ion [M+H]^+^ at *m*/*z* = 771 in positive mode. The fragmentation patterns, including ions at *m*/*z* = 625, 463, and 325, confirm the presence of glycosidic residues. Poliumoside is well-documented for its neuroprotective and anti-inflammatory properties [62], positioning *T. capitatum* as a promising candidate for further investigations in neurodegenerative diseases, such as Alzheimer’s disease [63].

Phenylethanoid glycosides, such as acteoside, were detected in significant quantities, especially in the hydroethanolic extract (5.29%). Acteoside displayed an ion [M-H]^−^ at *m*/*z* = 623, with distinctive pieces at *m*/*z* = 461, 179, and 161, validating its glycosidic structure. A prior study by Wu et al. [64] underscores the anti-inflammatory and immunomodulatory properties of phenylethanoid glycosides, hence enhancing the therapeutic potential of these extracts for inflammation-related disorders.

Phenolic acids, including chlorogenic acid and rosmarinic acid, were identified in significant amounts, especially in the decoction, where they constituted 13.01% of the overall composition. These chemicals are acknowledged for their strong antioxidant properties, especially in their capacity to impede lipid peroxidation and safeguard cells from oxidative damage [65]. Rosmarinic acid, in particular, has been the subject of numerous studies demonstrating its anti-inflammatory and antiviral properties [66].

Despite their lower concentrations, iridoids are of significant importance due to their pharmacological properties. Their minimal extraction in hydroethanolic solvents and superior recovery in aqueous solvents support the findings of Náthia-Neves et al. [67], who emphasized the significance of solvents in the extraction of polar chemicals like iridoids. These chemicals are well documented for their analgesic and anti-inflammatory properties, and their concentration in the decoction (8.36%) may elucidate some traditional applications of *T. capitatum* for mitigating pain and inflammation.

The selection of solvents profoundly influences the extraction efficacy of various substances. Methanolic and hydroethanolic solvents had superior efficacy in extracting flavonoids and phenylethanoid glycosides, but aqueous extraction was more appropriate for iridoids and vitamins. This conclusion corresponds with the findings of Durling et al. [68], who demonstrated that polar solvents, like methanol and ethanol, are more effective for extracting polyphenols, whereas aqueous solvents facilitate the preservation of water-soluble components like vitamins.

The presence of Vitamin C (ascorbic acid) at 3.17% and dehydroascorbic acid at 2.64% in the decoction substantiates the antioxidant capacity of the aqueous extract. These findings align with the documented function of vitamin C in neutralizing free radicals and safeguarding against oxidative stress [66].

Coumarins, particularly cirsilioline, exhibited a varied distribution depending on the extraction method, with the highest concentration observed in the hydroethanolic extract (4.81%). While coumarins are well known for their anticoagulant properties, which suggest potential applications in thrombosis prevention, it is important to note that some coumarins may exhibit hepatotoxic effects, warranting careful consideration of their use.

Sesquiterpenoids, including emmotin H, were predominantly identified in the decoction (1.11%), supporting the notion that water extraction is more efficient for isolating these compounds. Despite their modest abundance, sesquiterpenoids possess significant pharmacological properties, including anti-inflammatory and antibacterial activities [69].

Conversely, fatty acids were found in minimal amounts across all extracts, with the greatest quantity seen in the decoction (1.80%). While their impact on the overall bioactivity of the extracts may be minimal, their existence contributes to the intricacy of the chemical profile of *T. capitatum*.

The HPLC/UV-ESI-MS study of *T. capitatum* extracts identified a broad array of bioactive components, including flavonoids, phenylethanoid glycosides, and phenolic acids. The extraction strategy markedly affected the chemical makeup of the extracts, with polar solvents demonstrating superior efficacy in extracting polyphenols, while aqueous solvents facilitated enhanced recovery of iridoids and vitamins. These findings underscore the potential of *T. capitatum* as a source of bioactive compounds, with possible applications in the management of oxidative stress, inflammation, and neurodegenerative disorders. Future studies may concentrate on the in vitro and in vivo confirmation of the therapeutic effects of these substances, therefore substantiating the traditional use of *T. capitatum* in phytotherapy.

### 2.7. Evaluation of the Antioxidant Activity of the EOs and Extracts of T. capitatum

**Figure 9:**
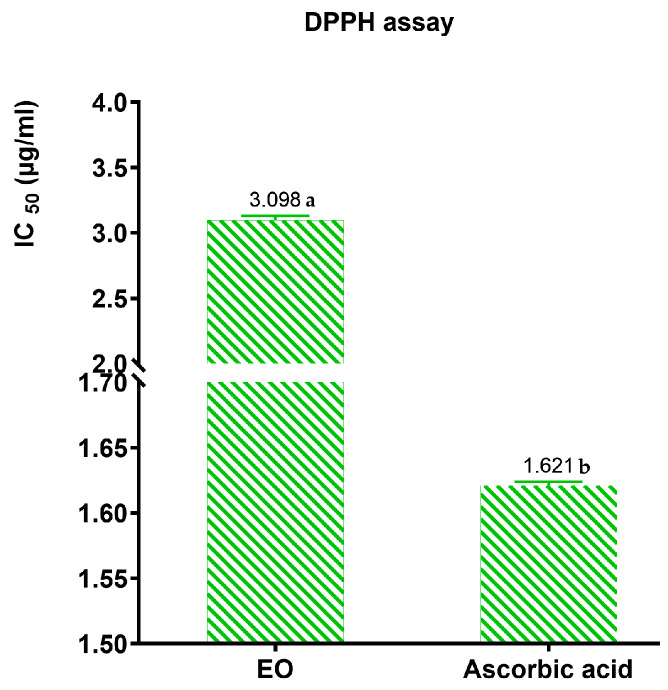
IC_50_ values of *T. capitatum* oil and the standard using the DPPH method. The results with different letters are significantly different from each other (*p* < 0.001).

The antioxidant potential of the EO of *T. capitatum* was evaluated in vitro using the DPPH method, with ascorbic acid as a positive control. The IC_50_ value of 3.098 μg/mL obtained for the EO indicates a significant antioxidant capacity (Figure 9), although it is lower than that of ascorbic acid (1.621 μg/mL). The EO of *T. capitatum* demonstrates promising potential, although slightly inferior to the standard. These findings highlight its noteworthy antioxidant properties.

Khaled-Khodja N. et al. [70] found an IC_50_ value of 95 μg/mL, studying the antioxidant effect of the EOs of *Teucrium polium* from (Bejaia, Algeria), which is higher than the antioxidant power of the EOs of the studied plant. As well as the results found by [47,71,72] of IC_50_ ranged from 17.56 to 23.6 μg/mL while the result found by Chabane et al. [52] is 5530 μg/mL. Concerning the work of Maizi et al. [73], they showed an IC_50_ equal to 16,000 ± 0.15 μg/mL. For *Teucrium polium geyrri*, the result found by Hammoudi R [74] is 19.46 μg/mL, despite being the same species. On the other hand, compared to other species such as *Teucrium marum* (Lamiaceae), Ricci, D., et al. [75] find an IC_50_ value of 13.13 μg/mL, and Bencheikh S. E. [76] obtains an IC_50_ of 58.6336 μg/mL. Therefore, we can say that the oil of *T. capitatum* has a very significant antioxidant power compared to the work carried out. The DPPH test demonstrated significant antioxidant activity. Additionally, the oil showed a strong capability to neutralize the DPPH radical. This significant biological impact is probably attributable to the chemical makeup of the EOs [77]. This low DPPH radical scavenging activity can be explained by their low content of phenolic compounds. The latter are known for their important roles as antioxidant agents [78,79,80]. It has been established in many studies that the antioxidant activity of EOs is not only related to the high percentage of major compounds, but also to the presence of other minor constituents and the synergistic effects between them [81,82,83,84,85,86,87,88].

**Figure 10:**
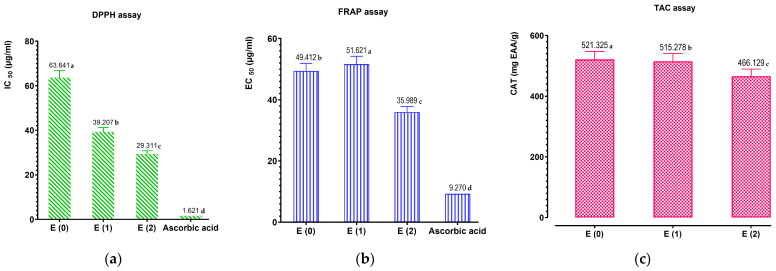
Antioxidant activity of ascorbic acid and extracts by: (**a**) DPPH; (**b**) FRAP; (**c**) TAC assays. Mean values ± standard deviations of determinations performed in triplicate are reported; means are significantly different (*p* < 0.001). The results with different letters are significantly different from each other (*p* < 0.001).

To evaluate the antioxidant properties of the aqueous, hydroethanolic, and hydromethanolic extracts of Teucrium capitatum, as well as a control (ascorbic acid), three techniques—DPPH, FRAP, and CAT—were used. The calibration curves for ascorbic acid were determined using the DPPH method (IC_50_: 1.621 ± 0.003 μg/mL) and the FRAP method (EC_50_: 9.270 ± 0.004 μg/mL). Recognized as natural antioxidants due to their ability to mitigate and/or prevent the formation of free radicals, these extracts were analyzed to compare their potential. As illustrated in Figure 10a, the results of the DPPH test reveal that the extracts of *T. capitatum* exhibit significant free radical scavenging activity. Notably, the hydromethanolic extract demonstrated the strongest antioxidant activity, with an IC_50_ value of 29.311 ± 1.465 μg/mL, compared to 39.207 ± 1.960 μg/mL for the hydroethanolic extract, and 63.641 ± 3.185 μg/mL for the aqueous extract. In comparison, ascorbic acid showed a significantly lower IC_50_ value of 1.621 ± 0.003 μg/mL. Furthermore, the results of the FRAP method, illustrated in Figure 10b, highlight significant differences in reducing power among the studied extracts and the positive control (ascorbic acid). The hydromethanolic extract exhibited the highest reducing power (35.989 ± 1.799 μg/mL), followed by the hydroethanolic extract (51.621 ± 2.581 μg/mL) and the aqueous extract (49.412 ± 2.470 μg/mL). These values remain significantly lower than that of ascorbic acid (9.270 ± 0.004 μg/mL), underscoring the high effectiveness of the control.

Figure 10c shows the total antioxidant capacity (TAC), measured in gallic acid equivalents. In this regard, the aqueous extract of *T. capitatum* exhibited the highest total antioxidant capacity (521.325 ± 26.324 mg GAE/g of extract), closely followed by the hydroethanolic extract (515.278 ± 25.276 mg GAE/g of extract), while the hydromethanolic extract showed a slightly lower value (466.129 ± 23.128 mg GAE/g of extract). These results highlight the notable antioxidant potential of *T. capitatum* extracts, with the hydromethanolic extract generally demonstrating superior efficacy in terms of free radical scavenging and reducing power. However, the aqueous extract showed a remarkable advantage in terms of total antioxidant capacity, emphasizing the varying effectiveness of different extraction methods and the importance of solvent choice in maximizing antioxidant activity.

Recent studies have further underscored the antioxidant properties of various Teucrium species, indicating that the extraction method significantly influences the antioxidant capacity of these plants. For instance, research by Albayrak et al. [89] demonstrated that hydroethanolic extracts of Teucrium polium exhibited superior antioxidant activity compared to methanolic and aqueous extracts, corroborating our findings on the efficacy of the hydromethanolic extract of *T. capitatum* [89]. This supports the notion that certain solvents can enhance the extraction of bioactive compounds, thereby increasing antioxidant activity. Additionally, the observed higher total antioxidant capacity in the aqueous extract aligns with the findings of similar studies where aqueous extractions yielded significant levels of phenolic compounds, contributing to overall antioxidant effects [43,90]. This highlights the need for optimizing extraction protocols to harness the full potential of plant extracts for therapeutic applications, particularly in combating oxidative stress-related diseases.

### 2.8. Evaluation of the Antimicrobial Properties of the EOs and Extracts of T. capitatum

The examination of the antibacterial and antifungal properties of the EOs from *T. capitatum* demonstrates significant findings, aligning with other prior investigations on essential oils derived from the *Teucrium* genus. Our research demonstrates that the essential oils of *T. capitatum* have considerable bacteriostatic and bactericidal efficacy against resistant bacterial strains, including *S. aureus* BLACT, *E. coli*, *E. cloacae*, and *K. pneumoniae*, with minimum inhibitory concentration (MIC) and minimum bactericidal concentration (MBC) values of 25 µg/mL (Table 8). These findings highlight the effectiveness of these EOs, while several investigations have shown elevated minimum inhibitory concentration values [39].

Recent studies, such as those by Ruiters et al. [91] and Benali et al. [92], have also reported similar antimicrobial activities in other *Teucrium* species, often attributed to the presence of phenolic and terpenic compounds. For instance, Benali et al. [92] demonstrated that the EOs of *T. africanum*, *T. kraussii*, and *T. trifidum* exhibited similar MIC values against *S. aureus* and *E. coli*, suggesting that the active compounds present in these EOs, such as monoterpenes and sesquiterpenes, play a crucial role in inhibiting microbial growth.

The results against fungal strains, specifically *C. albicans*, *C. tropicalis*, and *A. niger*, with MIC values between 3.13 and 12.5 µg/mL, support prior research indicating that the terpenoid richness in *Teucrium* EOs provides considerable antifungal efficacy.

The antibacterial potency of the EOs from *T. capitatum* may be ascribed to their volatile constituents, including β-pinene, α-pinene, α-cadinol, and germacrene D, which are effective against many pathogens in several investigations [93,94,95,96]. These chemicals are recognized for their capacity to break the cell membranes of microorganisms, consequently enhancing membrane permeability and inducing the release of cytoplasmic contents, finally resulting in cell death [97].

In contrast, the extracts obtained by decoction (E0) and by Soxhlet (E1 and E2) exhibit lower antimicrobial activity compared to the EOs, with MIC and MBC generally ranging between 25 and 50 µg/mL. The hydromethanolic extract (E2) demonstrates excellent antifungal activity, particularly against *C. parapsilosis* and *A. niger*. This difference could be attributed to the chemical composition of the extracts, which are potentially less concentrated in active compounds compared to the EOs. The literature indicates that aqueous or alcoholic extracts of plants may extract lipophilic compounds, such as monoterpenes and sesquiterpenes, less effectively, which could explain their reduced efficacy [98].

The extracts derived from decoction (E0) and Soxhlet extraction (E1 and E2) demonstrate diminished antibacterial efficacy relative to the EOs, with MIC and MBC typically falling between 25 and 50 µg/mL. The hydromethanolic extract (E2) has remarkable antifungal efficacy, especially against *C. parapsilosis* and *A. niger*. This disparity may be ascribed to the chemical nature of the extracts, which are likely less concentrated in active chemicals than the essential oils. The research suggests that aqueous or alcoholic plant extracts may be less successful in extracting lipophilic chemicals, such as monoterpenes and sesquiterpenes, which might account for their diminished potency [98].

The research conducted by Ruiters et al. [91] and Darabpour et al. [99] revealed that essential oils abundant in monoterpenes have enhanced antibacterial efficacy relative to aqueous or alcoholic extracts, a finding which is corroborated by our investigation. The synergistic impact among the many components in the EOs of *T. capitatum* may explain the observed high activity. Numerous studies indicate that the biological efficacy of EOs arises from the interaction of their components, even those present in minimal amounts, hence augmenting their total antibacterial capacity [100,101,102].

The EOs of *T. capitatum* exhibit remarkable antibacterial efficacy, especially antifungal properties, exceeding that of extracts derived from decoction or Soxhlet extraction methods. This effectiveness is mostly ascribed to its abundance of volatile chemicals, including terpenoids. Consistent with other research, our findings affirm that the Eos of *Teucrium* species exhibit significant therapeutic potential against resistant bacterial and fungal strains, thus paving the way for their incorporation into the formulation of natural antimicrobial agents.

### 2.9. In Silico Molecular Docking Study of Major Compounds from the EOs and Extracts of Teucrium capitatum

The molecular docking analysis conducted on the major compounds of the EOs and extracts of *T. capitatum* highlights their binding affinities with various antioxidant (5QJ2, 2CDU, 3NRZ), antibacterial (2VEG, 3NRZ), and antifungal (3GNS, 1EA1, 1IYL) target proteins. This comparative study is crucial for evaluating the biological potential of these compounds as inhibitors of these proteins. The more negative the obtained docking scores are indicates a stronger binding affinity, suggesting an increased likelihood of effective interaction between the compounds and the target proteins.

Among the compounds of the EOs, germacrene D, bicyclogermacrene, spathulenol, and α-cadinol exhibited moderate affinities towards the tested antioxidant, antibacterial, and antifungal proteins. In particular, α-cadinol demonstrated docking scores as low as −7.6 kcal/mol with certain proteins, indicating good affinity, close to the scores of native ligands (Table 9). Notably, it showed high affinity with antibacterial and antifungal proteins, suggesting interesting potential as an inhibitor of these targets. Other compounds, such as bicyclogermacrene and spathulenol, also stood out for their significant binding affinity to antibacterial and antifungal proteins, with scores ranging from −6.4 to −7.6 kcal/mol, similar to values observed for native ligands.

The major flavonoids from the extracts, such as apigenin-7-*O*-rutinoside, acteoside, and poliumoside, exhibited significantly lower docking scores, reflecting a much higher binding affinity. For instance, apigenin-7-*O*-rutinoside showed scores of −10.7 kcal/mol for the antioxidant protein 5QJ2 and −10.3 kcal/mol for the antibacterial protein 3NRZ, significantly surpassing the scores of native ligands. Similarly, poliumoside stands out with excellent scores (−11.1 kcal/mol for 5QJ2 and −9.0 kcal/mol for 2VEG), indicating strong potential as an inhibitor of antioxidant and antibacterial proteins.

#### 2.9.1. Interaction with Antioxidant Proteins

The investigation of molecular interactions between the components of EOs and extracts of *T. capitatum* and antioxidant proteins demonstrated varying, but nevertheless considerable, affinities (Table 10). The NADPH oxidase protein (PDB ID: 2CDU), crucial for neutralizing free radicals, exhibited modest interactions with germacrene D. This molecule establishes Pi-Alkyl interactions with residues PHE B:14 and LYS B:17, in addition to a Pi-Sigma bond with TYR B:62. Hydrophobic interactions are essential for stabilizing the ligand–protein complex, therefore improving the binding of germacrene D to the protein’s active site. Spathulenol interacts with the 2CDU protein via Pi-Alkyl bonds with residues HIS B:10, PRO A:432, and TYR B:62, in addition to a Pi-Donor hydrogen bond with PHE B:14. These diverse interactions enhance the stability of the complex, emphasizing the significance of hydrophobic and hydrogen-bonding interactions in ligand fixation. Concerning the protein 3NRZ, germacrene D and spathulenol have considerable affinities. Germacrene D mostly engages via alkyl bonds with the residues LYS L:792, LYS C:754, ILE L:596, PRO L:597, and VAL L:591. These hydrophobic interactions provide effective stability to the complex via the involvement of nonpolar side chains. Spathulenol exhibits a detrimental donor-donor interaction with the residue ARG I:37, potentially undermining the stability of the complex. Nonetheless, spathulenol may still establish a connection despite this adverse interaction, indicating a somewhat strong affinity for the 3NRZ protein. Poliumoside is distinguished by its capacity to engage more persistently with both proteins, 2CDU and 3NRZ, due to a range of interactions. Poliumoside makes several conventional hydrogen bonds with residues ARG L:344 and GLU B:51, in addition to carbon–hydrogen bonds with GLU B:166 and GLN B:428, in conjunction with the 2CDU protein. It also demonstrates a Pi-Anion contact with the residue GLU B:51 and hydrophobic Pi-Alkyl interactions with LYS B:334 and TYR B:333. The many interactions, which include electrostatic and hydrophobic forces, allow poliumoside to attain a significantly reduced binding energy, underscoring its potential as a strong antioxidant agent. For the 3NRZ protein, poliumoside demonstrated comparable interactions, further enhancing the stability of the complex via a network of varied and robust interactions.

#### 2.9.2. Interaction with Antibacterial Proteins

The antibacterial proteins derived from the EOs and extracts of *T. capitatum* showed diverse interactions (Table 11). The compounds of the essential oils and extracts of *T. capitatum* exhibited a range of notable interactions with antibacterial proteins, specifically dihydropteroate synthase (PDB ID: 2VEG) and enoyl-acyl carrier protein reductase (FabI) (PDB ID: 3GNS). The interactions differ according to the characteristics of the molecules examined, including terpenes like germacrene D and bicyclogermacrene, or flavonoids such as poliumoside, acteoside, and apigenin-7-*O*-rutinoside. Dihydropteroate synthase (PDB ID: 2VEG) demonstrated modest binding energies for germacrene D and bicyclogermacrene, of around −6.4 to −6.7 kcal/mol. Germacrene D mostly engages in Pi-Sigma and Pi-Alkyl interactions with residue PHE A:151, facilitating stability via hydrophobic forces. Bicyclogermacrene exhibits a diverse range of interactions, including standard hydrogen bonds with residues ARG A:282 and ASP A:201, along with Pi-Alkyl interactions with PHE A:206 and LYS A:237, which enhance ligand stability. These interactions, although stable, exhibit less diversity in comparison to flavonoids such as poliumoside, acteoside, and apigenin-7-*O*-rutinoside. Poliumoside engages in several interactions, including standard hydrogen bonds and Pi-Anion interactions with ASP A:175, exhibiting an improved capacity to stabilize the complex with a reduced binding energy of −9.0 kcal/mol. Acteoside forms a mix of hydrophilic and hydrophobic contacts, including standard hydrogen bonds and Pi-Sigma interactions, which enhance its strong affinity for the protein. Germacrene D and bicyclogermacrene mostly bind to enoyl-acyl carrier protein reductase (FabI) from staphylococcus aureus (PDB ID: 3GNS) via hydrophobic interactions. Germacrene D establishes alkyl-type connections with residues LEU A:148, ILE A:211, and PRO A:192, while bicyclogermacrene exhibits Pi-Alkyl interactions with PHE A:252, underscoring stabilization via hydrophobic interactions. Nonetheless, these interactions do not provide stability as robust as that reported with flavonoids. Poliumoside exhibits a remarkable array of interactions, including standard hydrogen bonds with ARG A:13 and ALA A:15, with Pi-Sigma and Amide-Pi stacked interactions, which promote the stability of the complex. Acteoside exhibits analogous interactions, including hydrogen bonds with ARG A:14 and ASN A:110, as well as Pi-Donor hydrogen bond interactions, which enhance the observed high affinity. Ultimately, apigenin-7-*O*-rutinoside establishes hydrogen bonds with ALA A:15 and SER A:121, in addition to Pi-Sigma interactions with THR A:147, hence enhancing the stability of the complex. Terpenes like germacrene D and bicyclogermacrene mostly demonstrate hydrophobic interactions, resulting in modest binding energies and restricted stability. Conversely, flavonoids like poliumoside, acteoside, and apigenin-7-*O*-rutinoside have reduced binding energies due to a blend of hydrophilic and hydrophobic interactions, underscoring their enhanced potential as antibacterial agents. The findings demonstrate the improved effectiveness of flavonoids in stabilizing complexes with target antibacterial proteins, making these compounds very interesting for therapeutic use.

#### 2.9.3. Interaction with Antifungal Proteins

In interactions with antifungal proteins, the compounds derived from *T. capitatum* showed superior efficacy compared to those from the essential oils (Table 12). The isolated compounds of *T. capitatum* exhibited notable interactions with antifungal proteins 1EA1 and 1IYL, showing differing affinities based on the molecular characteristics (terpenes and flavonoids). Flavonoids demonstrated stronger binding affinity and a greater range of interactions than the terpenes present in the essential oils. The cytochrome P450 14α-demethylase protein (PDB ID: 1EA1), implicated in sterol biosynthesis in fungi, demonstrated moderate binding energies of around −6.7 kcal/mol for the terpenes spathulenol and α-cadinol, derived from essential oils. Spathulenol predominantly interacts via hydrophobic alkyl and Pi-Alkyl interactions with residues CA A:401 and PHE A:193, therefore stabilizing the ligand–protein complex. Notably, α-cadinol establishes hydrophobic alkyl contacts with MET A:148, Pi-Alkyl interactions with TRP A:244, and a Pi–Sigma interaction with PHE A:193. The existence of an adverse acceptor–acceptor interaction with ASP A:229 constrains the stability of this complex. Conversely, flavonoids including poliumoside, apigenin-7-*O*-rutinoside, and acteoside exhibited much greater affinities for the 1EA1 protein, with binding energies of −9.3, −8.4, and −9.0 kcal/mol, respectively. Poliumoside forms a network of hydrophilic contacts, including standard hydrogen bonds with residues GLU A:213, GLU A:51, and ASP A:120, with Pi-Anion interactions with GLU A:213, hence facilitating the increased stability of the complex. Apigenin-7-*O*-rutinoside forms hydrogen bonds with residues ASN A:106, ASP A:120, and GLY A:142, supplemented by Pi-Sigma and Pi-T-shaped contacts, therefore enhancing the stability of the complex. In the case of the 1IYL protein, terpenes like spathulenol and α-cadinol demonstrated mostly hydrophobic interactions, accompanied by modest binding energies. Spathulenol establishes standard hydrogen bonds with LEU D:419 and ASN D:421, in addition to hydrophobic alkyl contacts with PRO D:217 and LEU D:216. Notably, α-Cadinol engages in Pi–Sigma interactions with PHE D:414, as well as Alkyl and Pi-Alkyl interactions with residues ILE D:431 and LYS D:436.

The flavonoids showed diverse interactions and enhanced affinity for the 1IYL protein. Poliumoside establishes hydrogen bonds with ASN D:416, GLU D:416, and LEU D:419, with hydrophobic Pi-Alkyl contacts with PRO D:219 and Pi-Sigma interactions with TYR D:418. The many interactions enable poliumoside to attain a binding energy of −9.4 kcal/mol, indicating superior stability of the protein complex. Apigenin-7-*O*-rutinoside establishes hydrogen bonds and engages in hydrophobic Pi-T-shaped and Pi-Stacked interactions with residues like PHE A:225, TYR A:307, and LEU A:337, underscoring its effectiveness. Acteoside forms hydrogen bonds with residues ASN D:416, GLY D:429, and LEU D:216, and engages in Pi-Sigma interactions with residue TYR D:418. Adverse Donor–Donor and Acceptor–Acceptor interactions exist; nevertheless, they do not substantially diminish the overall stability of the complex, which maintains stability with a binding energy of −9.0 kcal/mol. The flavonoids, including poliumoside, apigenin-7-*O*-rutinoside, and acteoside, exhibited varied interactions and strong affinity for the antifungal proteins 1EA1 and 1IYL. These interactions encompass hydrogen bonds, electrostatic interactions, and hydrophobic interactions, which enhance the stabilization of ligand-protein complexes and yield superior inhibitory efficacy compared to the terpenes present in essential oils, such as spathulenol and α-cadinol, whose interactions are predominantly restricted to hydrophobic contacts.

The comparative examination of molecular interactions between flavonoids and terpenes derived from *T. capitatum* demonstrates notable disparities in their antioxidant, antibacterial, and antifungal properties. Flavonoids, notably poliumoside, apigenin-7-*O*-rutinoside, and acteoside, have considerable binding affinity to target proteins such as NADPH oxidase (2CDU), due to their capacity to establish persistent hydrogen bonds with essential residues like Glu378 and Tyr383. These compounds have binding energies of −11.1 kcal/mol, exceeding those of established substances like quercetin. Their strong affinity and interaction ability with target proteins highlight the promise of flavonoids as powerful natural antioxidants. Concerning antibacterial action, terpenes like germacrene D and spathulenol exhibited modest affinities for dihydropteroate synthase (PDB ID: 2VEG), with binding energies between −6.4 and −6.8 kcal/mol. Flavonoids exhibited greater binding energies (−9.5 kcal/mol), exceeding both terpenes and natural ligands. In vitro studies indicate that terpenes exhibit more efficacy than isolated flavonoids, possibly due to their enhanced membrane penetrating capability, which enables them to effectively disrupt bacterial membranes [103,104,105]. Flavonoids, including poliumoside and apigenin-7-O-rutinoside, exhibited remarkable binding affinity to target proteins, including cytochrome P450 14α-demethylase (PDB ID: 1EA1), with binding energies of −9.3 kcal/mol. The measured values surpass those of terpenes such as spathulenol and α-cadinol, which demonstrated binding energies of around −6.7 kcal/mol. Terpenes have significant antifungal activity in vitro due to their capacity to disrupt fungal membranes, as shown by Raj et al. [106] and Nazzaro et al. [107]. The synergistic mixture of terpenes in essential oils seems to augment this action, yielding more efficiency than isolated flavonoids. The flavonoids from *T. capitatum* are notable for their robust ability to establish persistent connections with target proteins, offering potential antioxidant, antibacterial, and antifungal effects. Terpenes are notable for their capacity to penetrate membranes, chemical stability, and synergistic effects within essential oils, resulting in significant antibacterial activity. A synergistic strategy integrating both flavonoids and terpenes may use their advantages to create novel, potent natural antibacterial and antioxidant medicines.

## 3. Materials and Methods

### 3.1. Plant Material

**Figure 11:**
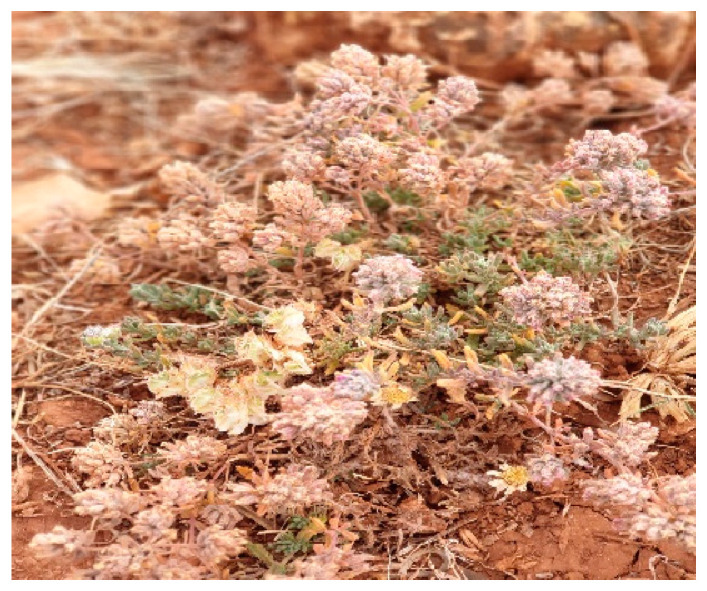
*T. capitatum* (Redouane Tarik and Touriya Zair, 2021).

In July 2021, the plant *T. capitatum* L. was harvested after its flowering period in the El Hajeb region, specifically in Ait Naaman (X = 5°20′20″ W; Y = 33°39′58″ N; Z = 558 m). Following collection, the plant was carefully dried at room temperature, and shielded from light, for two weeks to preserve its properties. Subsequently, it was formally identified by the Department of Botany at the Scientific Institute of Rabat. The leaves of the plant were selected for use in experimental procedures, providing a basis for the studies conducted. Comprehensive information regarding this species, including details relevant to the experimental work, is provided in Table 13 and Figure 11.

### 3.2. Microbial Material

The antimicrobial activity of extracts and essential oils (EOs) of *T. capitatum*. was evaluated on five bacterial strains (*Staphylococcus epidermidis*, *Staphylococcus aureus* BLACT, *Escherichia coli*, *Enterobacter cloacae*, and *Klebsiella pneumoniae*) and five fungal strains (*Candida albicans*, *Candida dubliniensis*, *Aspergillus niger*, *Candida tropicalis*, and *Candida parapsilosis*). These microorganisms were obtained from clinical samples taken from patients at the Mohamed V Provincial Hospital in Meknes, Morocco. The strains were initially preserved in a glycerin solution (20%) at −80 °C, then reactivated in Mueller–Hinton broth for bacteria and Sabouraud broth for fungi before subculturing.

### 3.3. Quality Control of Plant Material

#### 3.3.1. Determining pH

The acidity of the product in question is defined by its pH. The principle involves adding 10 mL of hot distilled water to a 2.0 g quantity of the sample under test. The mixture is filtered and then cooled. To measure the pH value, the electrode is then immersed in a large volume of this filtrate [108].

#### 3.3.2. Mineral Matter (Ash) and Organic Matter Content

The method is based on the calcination of a plant sample; 2.0 g of ground sample are placed in a muffle furnace at a temperature of 550 °C until all carbonaceous particles are destroyed, and a whitish ash of constant weight is obtained. The following Formula (1) is used to determine the organic matter content [108].

(1) $$MO\%=\left(\frac{M_{1}-M_{2}}{PE}\right)\times 100$$

where

MO% is the organic matter;

M_1_ is the weight of capsule and sample before calcination; 

M_2_ is the weight of capsule and sample after calcination; 

PE is the test sample.

Ash content is calculated as follows (2):

Ash% = 100 − MO%(2)

#### 3.3.3. Moisture Content of Dry Matter

The AFNOR standard (NF-V03-402 1985) is the method used [108]. The mass loss after 24 h of drying at 103 °C is used to calculate the moisture content. A 5 g sample is weighed, dried in a desiccator, and tared. Full crucibles are placed in an oven for 24 h at 103 °C. After this, they are weighed and cooled in a desiccator. According to the following relationship (3), the result is presented as a percentage of dry matter.

(3) $$TH\%=\left(\frac{M_{0}-M_{1}}{M_{0}}\right)\times 100$$

#### 3.3.4. Titratable Acidity

To determine the titratable acidity, 10.0 g of plant powder was added to 100 mL of boiling distilled water, stirred continuously for 15 min, and then filtered. From this, 10 mL of the filtrate was diluted with 20 mL of distilled water in a 100 mL beaker, and a few drops of phenolphthalein indicator were added. The acid solution was then titrated with a 0.01 N NaOH solution until a persistent pink color indicated the endpoint. The volume of NaOH used was recorded, and the titratable acidity was calculated as citric acid equivalents using the Formula (4) below [108,109]:

(4) $$Titratable\;acidity\left(\%\right)=\frac{Dilution\;factor\;*\;weight\;of\;acid\;equiv.\;*\;NaOH\;normality\;*\;titration\;vol.(mL)}{sample\;weight\;(g)}$$

Here, the “weight of acid equivalent” refers to the equivalent weight of citric acid used as a standard to express titratable acidity, allowing the acidity to be reported as a percentage of citric acid.

#### 3.3.5. Determination of Mineral Composition by ICP-AES

Trace metal, heavy metal, and trace element components were determined using inductively coupled plasma spectrometry (ICP/AES). This method provided a quick and accurate measurement of minor and major mineral content. One gram of powdered plant material was heated to 110 °C in a mixture of 5 mL concentrated nitric acid (HNO3) and 15 mL hydrochloric acid for mineralization. After complete solubilization, the sample was cooled to room temperature and diluted with ultra-pure water to a final volume of 100 mL. The solution was subsequently analyzed in duplicate at the UATRS (Technical Support Unit for Scientific Research) laboratory, where trace metal concentrations were measured directly using an ICP-AES spectrometer (Agilent 5110 ICP-AES) [110,111].

### 3.4. Phytochemical Study

Phytochemical screening is a qualitative method used to identify the chemical families within a plant. This process is based on reactions that either form insoluble complexes through precipitation or create colored complexes through staining [112,113,114,115]. The plant’s leaves and flowers were ground into a powder and subsequently sieved.

### 3.5. Extraction and Characterization of EO from T. capitatum

#### 3.5.1. EO Extraction

The EOs were extracted from the plant through hydro-distillation. For this purpose, 100 g of dried plant material was mixed with 1 L of water and distilled for 3 h at 90 °C in a flask equipped with a condenser and a Clevenger apparatus. As the plant material is heated, volatile compounds are released and carried away by steam. When the “water vapor volatile compounds” mixture passes through the condenser, it condenses into a two-phase solution. The EO, which typically has a lower density than water, is then separated, collected, and stored in a hermetically sealed brown glass bottle in a refrigerator at 4 °C.

#### 3.5.2. EO Content

The EO content (%EO) obtained is expressed as the volume of EO concerning the mass of dry matter (V/m). It is determined using the following Formula (5):

(5) $$\%\mathrm{EO}=\frac{V}{m}\;\times \;100$$

where

V is the volume of EO obtained (mL);

M is the mass of plant material used for hydrodistillation (g).

Oil quality control is essential to guarantee the safety of products intended for human consumption.

#### 3.5.3. Determining Density

The density of an EO at 20 °C is determined by the ratio between the oil’s density and that of pure water at the same temperature. It is calculated using the following Formula (6) [116].

(6) $$d_{20\;}=\frac{m_{2}-m_{0}}{m_{1}-m_{0}}$$

With 

m_0_: mass in grams of empty pycnometer

m_1_: mass in grams of pycnometer filled with water

m_2_: mass in grams of pycnometer filled with oil

### 3.6. Analysis and Identification of EO Chemical Composition by GC/MS

Gas chromatography combined with mass spectrometry (GC/MS) is used to perform chromatographic analysis of EO. The Thermo Electron chromatograph (Trace GC Ultra) is equipped with a DB-5 capillary column (5% phenyl-methyl-siloxane, 30 m × 0.25 mm; film thickness: 0.25 μm). The instrument is equipped with a split/splitless PVT (programmed spray temperature) injector. For injection, split mode is utilized with a leakage ratio of 1/70 and a flow rate of mL/min. The injected volume is 1 μL. The carrier gas (helium) has a flow rate of 1.5 mL/min, and the temperature programming has a gradient of 4 C/min. Mass spectrometry is performed using a Thermo Electron Trace MS system (Trace GC Ultra, Polaris Q). Fragmentation is performed by 70 eV electron impact. The system is connected to NIST 98, the mass spectra library of the National Institute of Standards and Technology. Kovats indices, calculated based on a hydrocarbon standard (C7-C40) injected under the same conditions, are used to identify HE compounds. Kovats indices are calculated using the following Formula (7) [117]:

(7) $$\mathrm{IK}=\left[\frac{\left({\mathrm{TR}}_{x}-{\mathrm{TR}}_{n}\right)}{\left({\mathrm{TR}}_{n+1}-{\mathrm{TR}}_{n}\right)}+n\right]\times \;100$$

where

IK is the Kovats index;

TR_x_ is the retention time of compound “x” to be identified;

n is the number of carbon atoms in the hydrocarbon eluted before compound “x”;

TR_n_ is the retention time of the hydrocarbon whose number of carbon atoms is “n”, and which is eluted before compound “x”;

TR_n+1_ is the retention time of the hydrocarbon with carbon number “n+1” eluted after compound “x”.

The calculated indices and mass spectra are then compared with those contained in available databases [118,119].

### 3.7. Extraction and Characterization of Phenolic Compounds from T. capitatum

To extract and characterize phenolic compounds from *T. capitatum*, two solid–liquid extraction methods were employed: Soxhlet extraction and decoction extraction.

**Soxhlet extraction** was performed using two different solvents: an ethanol/water mixture (7:3) and a methanol/water mixture (7:3). In each case, 300 mL of solvent was placed in a 500 mL flask, heated to 70 °C for hydroalcoholic mixtures and 100 °C for pure aqueous extracts. A cartridge containing 30 g of plant powder was inserted into the percolation column placed at the top of the flask, with a condenser to condense the vapors. After completing the extraction, the extracts were filtered and then concentrated to dryness under reduced pressure using a rotary evaporator, set to the appropriate temperature according to the solvent (70 °C for hydroalcoholic solutions, 100 °C for aqueous extracts). Finally, the resulting dry extract was weighed to calculate the extraction yield.

**Decoction extraction** involved adding 30 g of *T. capitatum* powder to 600 mL of distilled water. The mixture was heated on a hot plate and brought to a boil for one hour at a temperature of 80 °C. After this period, the solution was allowed to rest for five minutes before being filtered at low pressure. The resulting aqueous extracts were dried in an oven at 70 °C and then stored as a powder in glass vials until use. The extracts were coded as described in Table 14.

### 3.8. Extraction Yields

The following formula is used to calculate the extraction yields (Y) for both decoction and Soxhlet extraction (8):

(8) $$Y=\frac{m_{\mathrm{extract}}}{m_{0}}\;\times \;100$$

where

m_extract_ represents the mass of the effective components present in the dry matter after extraction, rather than the total weight of the extract, focusing only on substances that contribute to the extract’s efficacy;

m_0_ is the initial mass of the plant material used.

### 3.9. Quantification of Phenolic Compounds

#### 3.9.1. Determination of Phenolic Compound Content

To determine the content of phenolic compounds, including flavonoids, tannins, and total polyphenols (T), the following Formula (9) is used, ensuring that the measurement represents the effective phenolic components rather than the total extract weight:

(9) $$T=\frac{{C\cdot V}_{\mathrm{effective}}}{m}\;\times \;D$$

where

*C* denotes the mass concentration determined according to the calibration curve;

*V_effective_* is the volume of the extract that contains only the effective phenolic components;

M is the mass of the dry plant sample used for extraction;

D is the dilution factor, with D = V_f_/V_i_;

V_f_ is the final volume measured by spectrophotometry;

V_i_ is the volume taken from the extract for testing.

#### 3.9.2. Quantification of Total Polyphenols

The Folin–Ciocalteu technique, as reported by Singleton and Rossi [120], was used to determine the total polyphenol content of the plant extracts. This technique entails the oxidation of the polyphenols, followed by the production of color. The Folin–Ciocalteu reagent, composed of phosphotungstic (H_3_PW_12_O_40_) and phosphomolybdic (H_3_PMo_12_O_40_) acids, undergoes a reaction to produce blue tungsten oxides (W_8_O_23_) and molybdenum oxides (Mo_8_O_3_). These oxides are then measured using colorimetry at a wavelength of 760 nm. The absorbance was quantified using a UV mini-1240 spectrophotometer by comparing the samples to a blank, which consisted of a reaction mixture without the extract. Gallic acid was employed as a positive control, with concentrations varying from 50 µg/mL. A calibration curve was then established using the same experimental conditions. The results are reported in milligrams of gallic acid equivalents per gram of extract (mg GAE/g), which are determined using the equation derived from the calibration curve (Y = 0.095X + 0.003; R^2^ = 0.998). The experiment was conducted three times for each test.

#### 3.9.3. Quantification of Flavonoids

The measurement of flavonoid concentration was conducted using the colorimetric technique with aluminum trichloride, following the protocols described by Djeridane [50], Hung [121], and their collaborators. The compound aluminum chloride (AlCl_3_) undergoes a chemical reaction with the hydroxyl groups (OH) present in flavonoids, resulting in the formation of a complex. The quantification of flavonoids was conducted by using UV spectroscopy at a wavelength of 433 nm. The compound quercetin, a commonly used flavonoid, was subjected to identical analytical circumstances, with concentrations varying from 5 to 30 µg/mL. A calibration curve (Y = 0.073X − 0.081) was created using quercetin standards to measure the amounts of flavonoids in milligrams per gram of extract (mg/g). The experiment was replicated three times.

#### 3.9.4. Quantification of Condensed Tannins

The vanillin test [122] was employed to quantify the amounts of condensed tannins. Concisely, different quantities of a solution containing (+)-catechin (2 mg/mL) were introduced into 3 mL of a vanillin/methanol solution (4%; *w*/*v*). The liquid was stirred by hand, and then 1.5 mL of strong hydrochloric acid was added to each sample. The resultant mixes were subjected to a 20 min reaction at ambient temperature. Utilizing a UV-visible spectrophotometer, the absorbance at a wavelength of 499 nm was determined by comparing it to a blank sample. The condensed tannin concentration was quantified using a catechin-based calibration curve (Y = 0.7421X + 0.0318; R^2^ = 0.998) and expressed as milligrams of catechin equivalents per gram of extract (mg EC/g).

### 3.10. Quantitative Analysis of Polyphenols Was Conducted Using High-Performance Liquid Chromatography Coupled with Ultraviolet Detection and Electrospray Ionization Mass Spectrometry (HPLC/UV-ESI-MS)

The HPLC UltiMate 3000 system (Thermo Fisher Scientific, Sunnyvale, CA, USA), equipped with an autosampler, was utilized to analyze the phenolic compounds in *Teucrium* extracts through high-performance liquid chromatography coupled with mass spectrometry (HPLC/UV-ESI-MS) using exactive plus mass spectrometry and electrospray ionization. Samples were maintained at 5 °C. This HPLC system was fitted with a reverse-phase C18 column (250 × 4 mm, 5 μm particle size, Lichro CART, Lichrospher, Merck, Darmstadt, Germany). During analysis, the column temperature was set to 40 °C, and the mobile phase was degassed via ultrasonic treatment. Solvent A contained 0.1% formic acid in water, while solvent B comprised 0.1% formic acid in acetonitrile. The gradient composition started with 2% of solvent B (0 min), then increased to 30%, 95%, 2%, and 2% at 20, 25, 26, and 30 min, respectively. The injection volume was 20 μL, and the flow rate was maintained at 1 mL/min. Following negative ionization by electrospray, the detection was performed in MS/MS mode (broadband collision-induced dissociation, bbCID) using a Maxis Impact HD mass spectrometer (Bruker Daltonik, Bremen, Germany). Additionally, UV detection was conducted using an L-2455 diode array detector (Merck-Hitachi, Darmstadt, Germany), scanning within the wavelength range of 190–600 nm, with specific acquisitions at 280 nm, 320 nm, and 360 nm. Other parameters were defined as follows: capillary voltage at 3000 V; drying gas temperature at 200 °C; drying gas flow rate of 8 L/min; nebulizing gas pressure at two bars; and a deflection plate at −500 V. Nitrogen was used as the nebulizing and desolvation gas. MS data were collected across an *m*/*z* range of 50 to 750. Data acquisition and evaluation were conducted using the Thermo Scientific™ Chromeleon™ 7.2 Chromatography Data System (CDS).

### 3.11. Antioxidant Activities

#### 3.11.1. DPPH Test

The antioxidant activity of the EOs and extracts was evaluated using the DPPH* (2,2-diphenyl-1-picrylhydrazyl) free radical scavenging assay, according to the modified method of Brand-Williams et al. [123]. Different concentrations of each extract and the EOs were mixed with 2.8 mL of DPPH* solution dissolved in absolute ethanol (0.024 mg/mL). After 30 min of incubation at room temperature, the absorbance was measured at 515 nm using a spectrophotometer. Absolute ethanol was used as a negative control. Butylated hydroxytoluene (BHT), butylated hydroxyanisole (BHA), and ascorbic acid (vitamin C) served as reference compounds. The test was performed in triplicate, and the percentage inhibition (PI) was calculated using the following Formula (10) [124]:

(10) $$PI\%=\frac{A_{0}-A}{A_{0}}\times 100$$

where

PI denotes the percentage inhibition;

$A_{0}$ is the optical density of the free radical solution (DPPH) in the absence of the extract (negative control);

A is the absorbance of the free radical solution (DPPH) in the presence of the extract.

#### 3.11.2. Ferric Reducing Antioxidant Power (FRAP) Assay

The reducing power of the extracts was evaluated using the ferric reducing antioxidant power (FRAP) method, as described by Oyaizu [125]. Volumes of 0.5 mL of each extract, at concentrations ranging from 0.5 to 5 mg/mL, were mixed with 2.5 mL of phosphate buffer (0.2 M, pH 6.6) and 2.5 mL of potassium ferricyanide (K_3_Fe(CN)_6_) solution at 1%. The mixture was incubated at 50 °C for 20 min. Then, 2.5 mL of 10% trichloroacetic acid (TCA) solution was added, and the mixture was centrifuged at 3000 rpm for 10 min. The supernatant was collected and mixed with 2.5 mL of distilled water and 0.5 mL of 0.1% ferric chloride (FeCl_3_) solution. The absorbance of the solutions was measured at 700 nm. Ascorbic acid served as the standard, and results were expressed in terms of the effective concentration at 50% (EC_50_), corresponding to the concentration required to reduce 50% of the iron.

The extracts’ reducing ability was assessed using the ferric reducing antioxidant power (FRAP) approach, as outlined by Oyaizu [125]. Volumes of 0.5 mL of each extract, with concentrations between 0.5 and 5 mg/mL, were combined with 2.5 mL of phosphate buffer (0.2 M, pH 6.6) and 2.5 mL of a 1% potassium ferricyanide (K_3_Fe(CN)_6_) solution. The mixture was incubated at 50 °C for 20 min. Subsequently, 2.5 mL of 10% trichloroacetic acid (TCA) solution was added, and the mixture was centrifuged at 3000 rpm for 10 min. The supernatant was obtained and combined with 2.5 mL of distilled water and 0.5 mL of 0.1% ferric chloride (FeCl_3_) solution. The absorbance of the solutions was recorded at 700 nm. Ascorbic acid was used as the standard, and findings were reported as the effective concentration at 50% (EC_50_), indicating the concentration necessary to decrease iron by 50%.

#### 3.11.3. Determination of Total Antioxidant Capacity (TAC)

The total antioxidant capacity of the extracts was measured using the phosphomolybdenum reduction method by Prieto et al. [126]. This method is based on the reduction of molybdenum Mo (VI) to molybdate Mo (V) in the presence of antioxidants, forming a green phosphate/Mo (V) complex in an acidic medium. A volume of 0.3 mL of each extract was mixed with 3 mL of reagent solution, composed of 0.6 M sulfuric acid, 28 mM sodium phosphate, and 4 mM ammonium molybdate. The tubes were incubated at 95 °C for 90 min. After cooling, the absorbance of the solutions was measured at 695 nm. The total antioxidant capacity (TAC) was calculated as a percentage of inhibition using the following Formula (11):

(11) $$TAC\;(\%)=\frac{A_{extract}-A_{control}\;}{A_{blanc}}\times 100$$

where

A_extract_ is the absorbance of the sample;

A_control_ is the absorbance of the control (without extract);

A_blanc_ is the absorbance of the blank.

### 3.12. Antimicrobial Activity

Using 96-well microplates and the microdilution method, the minimum inhibitory concentration (MIC) of the EOs and extracts of *T. capitatum* was determined [127]. The MIC corresponds to the lowest concentration of EOs or extracts required to completely inhibit the growth of the tested microorganism during incubation, as indicated by the absence of visible growth to the naked eye. A series of dilutions were prepared from a stock solution of EOs or extracts dissolved in 10% DMSO to obtain concentrations ranging from 5 to 0.93 × 10^−2^ mg/mL. These dilutions were prepared with a final volume of 100 µL for each concentration in Sabouraud broth for fungi and Mueller–Hinton medium for bacteria. Then, 100 µL of microbial inoculum, at a final concentration of 10⁶ or 10⁴ CFU/mL for bacteria or fungi, respectively, was added at each stage of the dilution series. After 24 h of incubation at 37 °C, 10 µL of resazurin was added to each well to assess bacterial growth. The color changed from purple to pink after a second incubation at 37 °C for two hours indicated the presence of microbial growth. The MIC value was defined as the lowest concentration preventing the color change of resazurin. The growth and sterility controls were the eleventh and twelfth wells, respectively. The test was performed in duplicate for each EO and extract. Terbinafine, a reference antifungal, was dissolved in 10% DMSO after grinding to obtain a concentration of 250 mg/mL. 

The minimum bactericidal concentration (MBC) and minimum fungicidal concentration (MFC) were determined by taking 10 µL from each well with no apparent growth and spreading it onto Mueller–Hinton agar for bacteria or in Sabouraud broth for fungi, followed by incubation for 24 h at 37 °C. The lowest concentration resulting in a 99.99% reduction in CFU/mL compared to the control was considered the MBC or MFC.

### 3.13. Molecular Docking

The molecular docking methodology was employed to predict the interactions and binding affinities between the major compounds of the essential oils (EOs) and extracts of *T. capitatum* and selected target biomolecules. The proteins were specifically chosen based on their well-established roles in key biological processes targeted by the study. For antioxidant activities, PDB: 5QJ2, 2CDU, and 3NRZ were selected as they are enzymes or proteins involved in mitigating oxidative stress, such as free radical scavenging or antioxidant defense mechanisms. For antibacterial activities, PDB: 2VEG and 3NRZ were chosen due to their involvement in bacterial growth regulation or pathogenic mechanisms, serving as validated targets for antibacterial agents. For antifungal activities, PDB: 3GNS and 1EA1 were selected because they play crucial roles in fungal metabolism or cell wall integrity, which are key pathways targeted by antifungal compounds.

Detailed docking parameters and predicted binding sites are presented in Table 15, providing insights into the potential therapeutic properties of the bioactive compounds of *T. capitatum*. The selection of these proteins ensures relevance to the biological activities under investigation and facilitates an understanding of the specific interactions between *T. capitatum* compounds and these critical molecular targets.

Prior to docking, the protein structures underwent preprocessing steps to ensure their compatibility for analysis. Water molecules, heteroatoms (hetatm), unwanted chains, and co-crystallized ligands were removed. Polar hydrogen atoms and Gasteiger charges were added to the structures, which were subsequently converted into pdbqt format for docking. The three-dimensional crystal structures of the selected proteins were retrieved from the RCSB database in PDB format (https://www.rcsb.org; accessed 15 July 2024). Using PyMOL 2.3, non-essential residues and water molecules were removed, while nonpolar hydrogen atoms were added to maintain structural integrity. Energy minimization was then performed using Swiss PDB Viewer, and the optimized macromolecules were saved in PDB format for further analysis.

The docking simulations employed a semi-flexible modeling approach using PyRx AutoDock Vina. The target proteins were prepared as macromolecules, while the 3D conformers of the ligands, initially in SDF format, were imported and energy optimized. The ligands were then converted into pdbqt format using Open Babel. The docking simulations were conducted with a grid size of 40 Å × 40 Å × 40 Å, ensuring sufficient coverage of the active sites. The resulting docking outputs, including macromolecules and ligands, were exported in pdbqt format, merged, and saved in PDB format for visualization.

The docking results were analyzed using Discovery Studio Visualizer (version 4.6), which facilitated the generation of detailed 2D and 3D visualizations of the protein–ligand interactions. These findings provide a foundation for understanding the interaction mechanisms of *T. capitatum* compounds with target biomolecules, supporting their potential therapeutic applications.

## 4. Conclusions

This work emphasizes the considerable medicinal potential of *T. capitatum*, a member of the Lamiaceae family, using thorough chemical characterization, antibacterial and antioxidant assessments, and molecular docking investigations. The essential oils, rich in bioactive components including β-pinene and α-cadinol, have significant antibacterial and antioxidant properties. Furthermore, phenolic substances, such as poliumoside, enhance the potential antioxidant, and antimicrobial effects of the plant extracts. The in silico docking research elucidated the interaction processes of these principal compounds with biological targets, indicating their potential for future investigation in the creation of natural medicinal medicines. *T. capitatum* seems to be a significant source of bioactive chemicals applicable in the pharmaceutical, cosmetic, and nutraceutical sectors. Additional research is advised to validate these results and investigate the underlying processes in vivo. While this work underscores the significant antioxidant and antibacterial properties of *T. capitatum* extracts, the potential hepatotoxicity associated with the *Teucrium* genus warrants careful consideration. Although teucrins were not detected in this analysis, additional studies are necessary to definitively confirm their absence and assess the safety profile of *T. capitatum* for therapeutic use.

## Figures and Tables

**Table 1:** Quality control of the plant material of *T. capitatum*.

MC (%)	pH	Acidity	Ashes
10.02 ± 1.69	5.89 ± 0.01	0.33 ± 0.01	87.80 ± 0.11

**Table 2:** Heavy metal contents in the plant *T. capitatum*.

Element	Heavy Metal Content (mg/L)
Arsenic (As)	0.0976
Zinc (Zn)	1.688
Cobalt (Co)	<0.0001
Manganese (Mn)	1.231
Iron (Fe)	7.414
Copper (Cu)	0.3437
Aluminum (Al)	4.246

**Table 3:** Phytochemical screening of *T. capitatum* leaves.

Chemical Groups			Observations
Primary metabolites	Polysaccharides		++
	Lipids		+++
	Proteins	Biuret reaction	−
		Xanthoproteic reaction	+++
	Reducing sugars		+
Secondary metabolites	Tannins	Gallic	++
		Catechic	+++
	Flavonoids	Flavones	++
		Leucoanthocyanins	++
	Anthocyanins		−
	Saponins		+
	Alkaloids	Mayer	−
		Dragendorff	−
		Wagner	−
	Reducing compounds		++
	Oses and holosides		++
	Mucilages		++
	Sterols and triterpenes		++

+++ Very abundant; ++: abundant; + weak; − absent.

**Table 4:** Organoleptic and physicochemical properties of *T. capitatum* EOs.

Density	Color	Odor
0.962 ± 0.004	Yellow-orange	Strong and pronounced

**Table 5:** Chemical composition of the EOs of *T. capitatum*.

N°	Compound	KI (Adams)	RA (%)	Formula
1	α-pinene	939	6.55	C_10_H_16_
2	β-pinene	979	24.50	C_10_H_16_
3	Myrcene	990	0.54	C_10_H_16_
4	Limonene	1029	4.83	C_10_H_16_
5	trans-Pinocarveol	1139	0.60	C_10_H_16_O
6	Nopinone	1140	0.39	C_9_H_14_O
7	Myrtenal	1195	0.42	C_10_H_14_O
8	α-Terpineol	1188	0.53	C_10_H_18_O
9	γ-Elemene	1436	0.65	C_15_H_24_
10	β-Bourbonene	1388	0.31	C_15_H_24_
11	(E)-Caryophyllene	1419	1.94	C_15_H_24_
12	(E)-β-Farnesene	1456	0.39	C_15_H_24_
13	α-Humulene	1454	0.30	C_15_H_24_
14	Dehydro-Sesquicineole	1471	1.32	C_15_H_24_O
15	Germacrene D	1481	4.94	C_15_H_24_
16	Bicyclogermacrene	1500	4.67	C_15_H_24_
17	α-Muurolene	1500	0.39	C_15_H_24_
18	γ-Cadinene	1513	0.82	C_15_H_24_
19	δ-Cadinene	1523	3.08	C_15_H_24_
20	Caryophyllene oxide	1583	0.30	C_15_H_24_O
21	Germacrene D-4-ol	1575	1.11	C_15_H_26_O
22	Spathulenol	1578	3.13	C_15_H_24_O
23	Viridiflorol	1592	1.39	C_15_H_26_O
24	Allo-Aromadendrene epoxide	1641	0.37	C_15_H_24_O
25	Cubenol	1646	0.64	C_15_H_26_O
26	epi-α-Cadinol	1640	4.42	C_15_H_26_O
27	α-Cadinol	1654	17.02	C_15_H_26_O
28	Bulnesol	1671	0.56	C_15_H_26_O
29	epi-α-Bisabolol	1684	0.90	C_15_H_26_O
30	Shyobunol	1689	12.13	C_15_H_26_O
31	(2Z,6E)-Farnesol	1723	0.48	C_15_H_26_O
Compounds identified (%)		99.62		
Sesquiterpenes (%)		17.83		
Oxygenated sesquiterpenes (%)		43.43		
Monoterpenes (%)		36.42		
Oxygenated monoterpenes (%)		1.94		

**Table 6:** Compounds Identified by HPLC/UV-ESI-MS in *T. capitatum* Extracts.

RT	Molecules	Structure	Classes	Exact Weights	[M−H]^−^ (*m*/*z*)	[M+H]^+^ (*m*/*z*)	Fragment Ions (*m*/*z*)	Area (%)		
								E (0)	E (1)	E (2)
3.91	Embelic acid	C_17_H_26_O_4_	Fatty acid	294	293		293-184	1.03	0	0.15
4.06	Ferulic acid	C_10_H_10_O_4_	Phenolic acid	194		195	178-149-134	0.42	0	0.11
4.66	Salvigenin	C_18_H_16_O_6_	Flavonoid	328		329	314	0.65	1.72	0.21
4.78	Luteolin 7-*O*-(6′-malonylglucoside)	C_24_H_22_O_14_	Flavonoid	534	533		447-285-253	0.39	0	0
4.94	Azelaic acid	C_9_H_16_O_4_	Fatty acid	188	187		169-125	0.39	0.82	0
5.13	Myricetin	C_15_H_10_O_8_	Flavonoid	318	317		317-287-178	0.52	1.09	1.52
6.77	Coumarin	C_9_H_6_O_2_	Coumarin	146		147	147-103	1.29	1.53	0.27
7.14	Linocaffein	C_15_H_18_O_9_	Phenolic compound	342	341		179-135-117	0	0	0.25
9.6	trans-caftaric acid	C_13_H_12_O_9_	Phenolic acid	312	311		249-203-179-149		1.31	0
9.89	Pimelic acid	C_7_H_12_O_4_	Fatty acid	160	159		115-97	0.38	0	0
9.89	Quinic acid	C_7_H_12_O_6_	Phenolic acid	192	191		191-147	0.77	1.13	0.66
9.92	Hydroxycaffeic acid	C_9_H_8_O_5_	Phenolic acid	196	195		179-135	0	0.41	0
10.24	Swertiapunimarin	C_22_H_32_O_14_	Iridoid	520	519		477-315-271	0	0	0.19
10.64	Cyasterone	C_29_H_44_O_8_	Others	520	519		479-457-371	1.45	0	0
10.99	Dihydromyricetin	C_15_H_12_O_8_	Flavonoid	320		321	303-183-165-139	0	0	0.41
11.51	Oleuropein aglycone	C_19_H_22_O_8_	Coumarin	378	379		197-158	2.05	3.28	0.82
11.91	Dehydroascorbic acid	C_6_H_6_O_6_	Vitamin	174	173		143-125	2.64	0	0
12.65	p-Coumaroyl tartaric acid	C_13_H_12_O_8_	Phenolic compound	296	295		163-135-119	0	0	0.71
13	Dihydrosamidin	C_21_H_24_O_7_	Coumarin	388	387		343-223-197	0.74	0	0.22
13.37	Schizonepetoside E	C_16_H_28_O_8_	Phenylethanoid glycoside	348		349	331-289-151	0.41	0	0
14.8	Eriodictyol	C_15_H_12_O_6_	Flavonoid	288		289	247-179-163	0	1.15	8.14
14.92	Rosmarinic acid	C_18_H_16_O_8_	Phenolic acid	360		361	181-163-145-135-117	1.77	0.68	2.3
15.48	Esculin	C_15_H_16_O_9_	Coumarin	340	339		177-133	0	0	0.15
15.57	1-Caffeoyl-*β*-D-glucose	C_15_H_18_O_9_	Phenolic compound	342	341		179-135-119	0	1.19	0
15.84	Oleuropeinic acid	C_25_H_30_O_15_	Phenolic compound	570	569		307-225-137	1.57	0	0
16.1	3,4-Dimethylumbelliferone	C_11_H_10_O_3_	Coumarin	190	189		145-117	0	0	0.16
16.18	Catechin hydrate	C_15_H_16_O_7_	Flavonoid	308	309		289-245-179	0	1.23	0
16.67	Kaempferol 3-*O*-rutinoside	C_27_H_30_O_15_	Flavonoid	594		595	449-287	3.73	3.04	0.37
17.03	Ascorbic acid	C_6_H_8_O_6_	Vitamin	176	175		175-115-87	1.52	0	0
17.3	2-O-caffeoylglucaric acid	C_15_H_16_O_11_	Phenolic acid	372	371		179-135-117	1.62	0	0.66
17.76	Luteolin 7-diglucuronide	C_27_H_26_O_18_	Flavonoid	638		639	463-287-141	0.18	0	0
18.08	Syringic acid	C_9_H_10_O_5_	Phenolic acid	198	197		179-135-123	0.96	2.22	0.18
18.77	Harpagide	C_15_H_24_O_10_	Iridoid	364	363		345-183-165	3.74	0	0.21
19.12	Calcitriol	C_27_H_44_O_3_	Vitamin	416	415		397-355-289	0.21	0	0
19.37	Ligstroside aglycone	C_19_H_22_O_7_	Phenolic compound	362	361		197-153-137	0	0.7	3.2
19.4	Chicoric acid	C_22_H_18_O_12_	Phenolic acid	474	473		311-293-179-149	3.17	0.36	0.42
19.6	Emmotin H	C_15_H_16_O_3_	Sesquiterpenoid	244	243		243-228-200-184	1.11	0	0
19.81	Cianidanol	C_15_H_14_O_6_	Flavonoid	290		291	165-139-123	0.55	1.29	0
19.82	Poliumoside	C_35_H_46_O_19_	Phenylethanoid glycoside	770		771	625-463-325	15.28	18.47	27.74
20.12	Trans-p-coumaric acid 4-glucoside	C_15_H_18_O_8_	Phenolic compound	326	325		163-119	0	1.31	0
20.21	Centauroside	C_34_H_46_O_19_	Iridoid	758		759	597-455-325	0.97	0	0
20.3	Naringenin	C_15_H_12_O_5_	Flavonoid	272		273	153-119	0	0	0.63
20.7	Feruloyltartaric acid	C_14_H_14_O_9_	Phenolic compound	326	325		193-149-134	2.71	0	0.82
21.47	Loganic acid	C_16_H_24_O_10_	Iridoid	376		377	162-85	0.41	0	0
21.92	Arctigenin	C_21_H_24_O_6_	Others	372		373	305-237-177-137	0	1.07	0
21.98	Cirsiliol	C_17_H_14_O_7_	Flavonoid	330	329		329	1.35	7.82	16.18
22.37	Diosmetin 7-*O*-rutinoside	C_28_H_32_O_15_	Flavonoid	608	607		607-341-299	0.42	0.69	1.72
22.57	Acteoside	C_29_H_36_O_15_	Phenylethanoid glycoside	624	623		623-461-179-161	0	5.29	2.89
22.92	Mangiferin	C_19_H_18_O_11_	Flavonoid	422		423	423-273	3.27	0	1.05
23.75	Chlorogenic acid	C_16_H_18_O_9_	Phenolic acid	354	353		191-179	4.3	0	
24.46	Apigenin-7-*O*-rutinoside	C_27_H_30_O_14_	Flavonoid	578		579	579-269	5.04	5.32	5.7
24.49	Apigenin 7-O-glucoside	C_21_H_20_O_10_	Flavonoid	432	431		431-269	0	3.24	2.3
24.59	Rutin	C_27_H_30_O_16_	Flavonoid	610	609		609-301	0.64	1.09	0
25.6	p-Coumaric acid	C_9_H_8_O_3_	Phenolic compound	164		165	147-119	0.5	0	0
25.79	Naringenin-7-O-glucoside	C_21_H_22_O_10_	Flavonoid	434	433		433-271	0	15.93	0
26.11	Acacetin	C_16_H_12_O_5_	Flavonoid	284	283		283-243	0	0	7.76
26.4	7-Methoxy-2-methylisoflavone	C_17_H_14_O_3_	Flavonoid	266	265		250-223	0	0.89	0
26.46	Kaempferol	C_15_H_10_O_6_	Flavonoid	286		287	287-165-153	0	1.85	0
26.5	Cirsimaritin	C_17_H_14_O_6_	Flavonoid	314		315	254-226-136	28.22	2.81	3.51
26.65	Secoxyloganin	C_17_H_24_O_11_	Iridoid	404	403		371-165-101	3.24	0	0
26.92	Embelin	C_17_H_26_O_4_	Others	294	293		293-184	0	1.02	0
27.08	Rhamnetin	C_16_H_12_O_7_	Flavonoid	316	315		300-179-165-151	0	4.4	0
27.62	Kaempferide	C_16_H_12_O_6_	Flavonoid	300	299		217-149-107	0	2.54	0
27.67	Cirsilineol	C_18_H_16_O_7_	Flavonoid	344	343		328-299-271	0.95	0	7.52
28.12	Diosmetin	C_16_H_12_O_6_	Flavonoid	300	299		299-285	0	0	0.53
28.18	Apigenin	C_15_H_10_O_5_	Flavonoid	270	269		227-159-121-105	0	2.46	0
28.85	Phenethyl cinnamate	C_17_H_16_O_2_	Others	252		253	253-131	0	0.55	0.27

**Table 7:** Distribution of chemical constituents in the EOs of *Teucrium capitatum*.

Classes of Compounds/Extracts	Area (%)		
	E(0)	E(1)	E(2)
Coumarin	3.34	4.81	1.62
Others	1.45	2.64	0.27
Sesquiterpenoid	1.11		
Vitamin	4.37		
Fatty acid	1.80	0.82	0.15
Phenolic acid	13.01	6.11	4.33
Phenolic compound	4.78	3.20	4.98
Flavonoid	45.91	58.56	57.55
Phenylethanoid glycoside	15.69	23.76	30.63
Iridoid	8.36	0.00	0.40
Total (%)	99.82	99.90	99.93

**Table 8:** The MIC, MBC, and MFC (µg/mL) of *T. capitatum* EOs and various extracts, along with the MIC of antibiotics and antifungal agents.

Microorganism		EO		E (0)		E (1)		E (2)		Antibiotics *				Antifungals ^#^
		MIC	MBC or MFC	MIC	MBC or MFC	MIC	MBC or MFC	MIC	MBC or MFC	Gentamycin	Amoxicillin–Clavulanate	Vancomycin	Trimethoprim–Sulfamethoxazole	Terbinafine
GPC	*S. epidermidis*	25	50	>50	>50	50	50	50	50	2		>8	>4/76	
	*S. aureus BLACT*	25	25	>50	>50	25	50	50	50	<0.5		2	<10	
GNB	*E. coli*	25	25	>50	>50	50	50	>50	>50	2	8/2		≤1/19	
	*E. cloacae*	25	25	>50	>50	50	50	50	50	>4	>8/2		>4/76	
	*K. pneumoniae*	25	25	>50	>50	>50	>50	50	50	≤1	≤2/2		≤1/19	
Yeasts	*C. albicans*	6.25	12.5	>50	>50	25	50	>50	>50					12.500
	*C. parapsilosis*	6.25	12.5	>50	>50	50	50	12.5	12.5					6.250
	*C. tropicalis*	6.25	12.5	>50	>50	50	50	25	50					12.500
	*C. dubliniensis*	6.25	12.5	>50	>50	25	25	25	50					3.125
Molds	*A. niger*	3.13	6.25	50	50	25	50	3.13	3.13					3.125

*: the MIC (µg/mL) of the antibiotics was determined by the BD Phoenix™ identification and antibiogram instrument; ^#^: the MIC (µg/mL) of terbinafine was determined on a microplate; GPC: Gram-positive Cocci; GNB: Gram-negative Bacill.

**Table 9:** Docking scores of *T. capitatum* L. EOs compounds with various target proteins (energies in kcal/mol).

	Antioxidant Proteins (PDB IDs)			Antibacterial Proteins (PDB IDs)		Antifungal Proteins (PDB IDs)	
**No Compounds**	5QJ2	2CDU	3NRZ	2VEG	3GNS	1EA1	1IYL
	Free binding energy [kcal·mol^−1^]						
**ligand natif**	−6.8	−7.7	−6.7	−6.9	−6.1	−6.9	−6
**α-Pinene**	−5	−6.4	−6.4	−5.1	−5	−5	−5.7
**β-Pinene**	−4.9	−6.4	−6.2	−5.3	−4.9	−5.1	−5.7
**Limonene**	−5.4	−6.5	−6.5	−5	−4.7	−5.4	−5.9
**Germacrene D**	−6.4	−8	−6.8	−5.7	−5.8	−6.6	−6.5
**Bicyclogermacrene**	−6.7	−7.6	−6.5	−6.4	−6.4	−6.4	−7.2
**epi-α-Cadinol**	−6.5	−7.5	−7.6	−5.9	−5.9	−6.5	−5.7
**α-Cadinol**	−6.6	−7.6	−7.6	−5.9	−5.9	−6.9	−6
**Shyobunol**	−6	−7.2	−6.1	−5.6	−5.3	−5.6	−5.5
**Spathulenol**	−6.8	−7.6	−6.8	−6.2	−6.3	−6.7	−6.4
**δ-Cadinene**	−6.3	−8	−8	−5.8	−6.1	−6.6	−6.7
**Acacetin**	−8.3	−7.8	−8.7	−7.2	−7.0	−7.7	−7.1
**Acteoside**	−8.6	−8.6	−9.5	−8.1	−8.5	−9.0	−7.7
**Apigenin-7-*O*-rutinoside**	−10.7	−8.3	−10.3	−8.8	−9.0	−8.4	−8.6
**Cirsilineol**	−8.7	−7.6	−9.0	−6.9	−7.5	−8.0	−6.7
**Cirsiliol**	−8.6	−7.6	−8.9	−6.9	−7.3	−7.8	−6.7
**Cirsimaritin**	−8.2	−7.8	−8.3	−6.6	−7.1	−7.7	−6.5
**Eriodictyol**	−8.7	−8.2	−8.1	−7.3	−7.1	−7.9	−7.1
**Naringenin-7-*O*-glucoside**	−9.4	−7.7	−9.3	−7.9	−8.2	−8.1	−8.1
**Poliumoside**	−11.1	−9.8	−10.0	−9.0	−9.2	−9.3	−9.4

**Table 10:** 2D and 3D interactions of compounds from the EOs and extracts of *T. capitatum* with target proteins.

	2CDU		3NRZ	
	2D	3D	2D	3D
**Germacrene D**	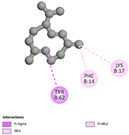	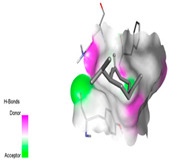	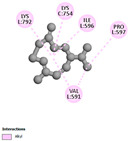	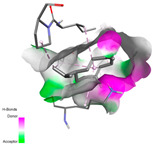
**Spathulenol**	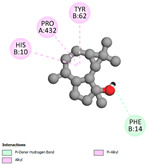	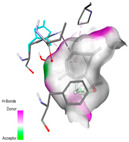	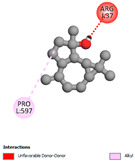	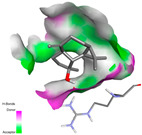
**Poliumoside**	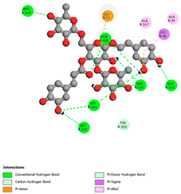	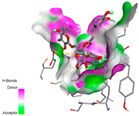	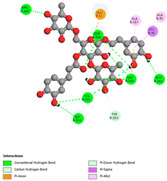	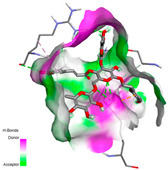

**Table 11:** 2D and 3D interactions of compounds from the EOs and extracts of *T. capitatum* with target proteins 2VEG and 3GNS.

	2VEG		3GNS	
	2D	3D	2D	3D
**Germacrene D**	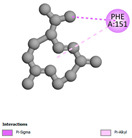	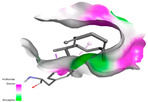	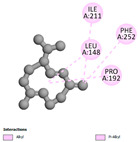	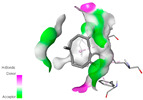
**Bicyclogermacrene**	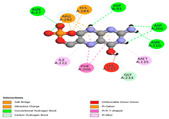	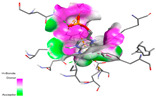	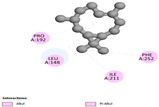	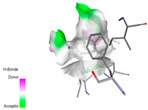
**Poliumoside**	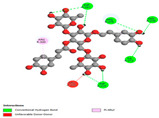	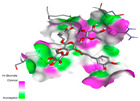	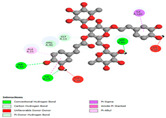	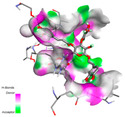
**Acteoside**	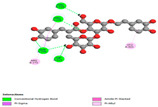	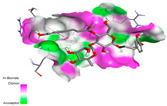	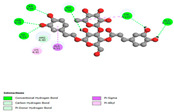	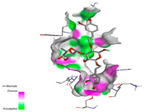
**Apigenin-7-*O*-rutinoside**	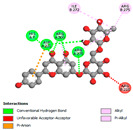	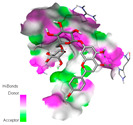	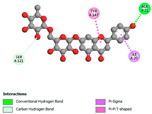	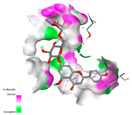

**Table 12:** 2D and 3D interactions of compounds from the EOs and extracts of *T. capitatum* with target proteins 1EA1 and 1IYL.

	1EA1		1IYL	
	2D	3D	2D	3D
**Spathulenol**	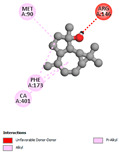	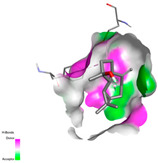	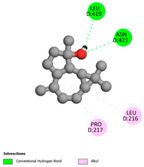	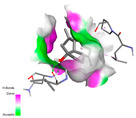
**α-Cadinol**	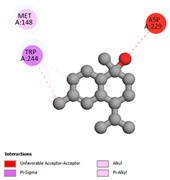	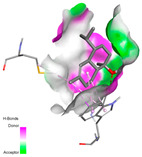	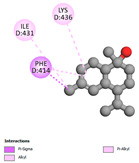	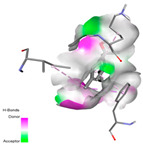
**Poliumoside**	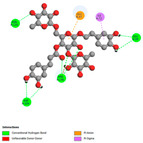	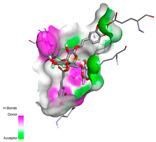	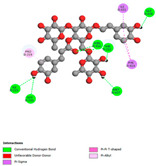	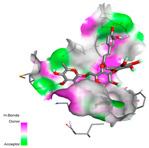
**Apigenin-7-O-rutinoside**	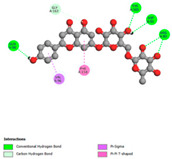	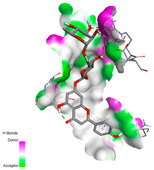	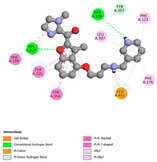	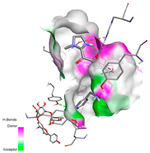
**Acteoside**	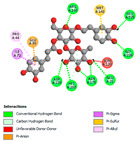	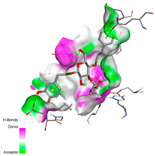	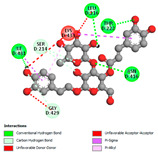	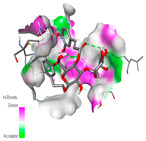

**Table 13:** Systematics of the plant *T. capitatum*.

Kingdom	Plantae
Sub-Reign	Viridaeplantae
Division	Magnoliophyta
Class	Equisetopsida
Subclass	Magnoliidae
Super-Order	Asteranae
Order	Lamiales
Family	Lamiaceae
Genus	*Teucrium* L
Species	*T. capitatum* L.

**Table 14:** Extraction coding.

Extraction Methods	Solvents	Codification
**Soxhlet**	Methanol/water (70/30; *v*/*v*)	E (2)
	Ethanol/water (70/30; *v*/*v*)	E (1)
**Decoction**	Water	E (0)

**Table 15:** Target protein parameters and molecular docking.

Protein	PDB ID	Grid Box Center Coordinates
Copper amine oxidase	5QJ2	Center_x = − 45.944 Center_y = 9.61 Center_z = 29.488
Phospholipase A2	2CDU	Center_x = 10.73 Center_y = 4.992 Center_z = 25.804
Carbonic anhydrase II	3NRZ	Center_x = 60.506 Center_y = 1.675 Center_z = 31.662
Dihydropteroate synthase	2VEG	Center_x = 38.872 Center_y = 57.261 Center_z = 16.831
*Β*-lactamase	3GNS	Center_x = − 14.28 Center_y = 0.562 Center_z = - 21.462
Human purine nucleoside phosphorylase	1EA1	Center_x = 13.26 Center_y = − 1.996 Center_z = 16.902
Cytochrome P450 2C9	1IYL	Center_x = 24.522 Center_y = 29.725 Center_z = 14.604

## Data Availability

The data are contained within this article.
